# Stapled peptide targeting the CDK4/Cyclin D interface combined with Abemaciclib inhibits KRAS mutant lung cancer growth

**DOI:** 10.7150/thno.40971

**Published:** 2020-01-12

**Authors:** Celine Bouclier, Matthieu Simon, Guillaume Laconde, Morgan Pellerano, Sebastien Diot, Sylvie Lantuejoul, Benoit Busser, Laetitia Vanwonterghem, Julien Vollaire, Véronique Josserand, Baptiste Legrand, Jean-Luc Coll, Muriel Amblard, Amandine Hurbin, May C. Morris

**Affiliations:** 1 Institut des Biomolécules Max Mousseron, CNRS, UMR 5247, Université de Montpellier, Faculté de Pharmacie, 15, Av. Charles Flahault, 34093 Montpellier, France; 2 CHU Grenoble Alpes, Université Grenoble Alpes, Grenoble, France; 3 Institut pour l'Avancée des Biosciences, INSERM U1209, CNRS UMR-5309, Université Grenoble Alpes, Grenoble, France

**Keywords:** CDK4, Stapled Peptide, Inhibitor, Lung cancer (NSCLC), *KRAS* mutation

## Abstract

CDK4/cyclin D kinase constitutes an attractive pharmacological target for development of anticancer therapeutics, in particular in *KRAS*-mutant lung cancer patients, who have a poor prognosis and no targeted therapy available yet. Although several ATP-competitive inhibitors of CDK4 have been developed for anticancer therapeutics, they suffer from limited specificity and efficacy.

**Methods**: As an alternative to ATP-competitive inhibitors we have designed a stapled peptide to target the main interface between CDK4 and cyclin D, and have characterized its physico-chemical properties and affinity to bind cyclin D1.

**Results**: We have validated a positive correlation between CDK4/cyclin D level and *KRAS* mutation in lung cancer patients. The stapled peptide enters cells rapidly and efficiently, and inhibits CDK4 kinase activity and proliferation in lung cancer cells. Its intrapulmonary administration in mice enables its retention in orthotopic lung tumours and complete inhibition of their growth when co-administered with Abemaciclib.

**Conclusion**: The stapled peptide targeting the main interface between CDK4 and cyclin D provides promising therapeutic perspectives for patients with lung cancer.

## Introduction

Lung cancer is the leading cause of cancer-related death worldwide in men and women 1. Non-small-cell lung carcinoma (NSCLC) accounts for 85% of all lung cancer cases, and includes adenocarcinoma (ADC) representing half of lung cancers and squamous cell carcinoma (SCC) (nearly 30%). Standard radiotherapy and chemotherapy were the only alternative, until the recent discovery of “driver” oncogenic mutations in a subset of adenocarcinomas and the development of corresponding targeted therapies, however mainly limited to patients harbouring the targeted genetic aberration 2.

CDKs are heterodimeric protein kinases formed through association of a CDK catalytic subunit with a cyclin regulatory partner 3, 4. CDK4 complexed with cyclin D isoforms, constitutes an established pharmacological target in several human cancers, associated with mutation of CDK4, amplification of cyclin D or overexpression of p16^INK4a^, all of which lead to hyperactivation of this kinase. CDK4/cyclin D activity coordinates exit from quiescence and growth factor-stimulated entry into and progression through G1 through phosphorylation of the Retinoblastoma tumour suppressor proteins (pRb, p107, p130). Cyclin D expression and its association with CDK4 are induced by mitogenic signals, notably via Ras signaling pathway. Amplification of the cyclin D1 locus is observed in 5-30% of NSCLC and high levels of cyclin D1 protein are found in 18-76% of invasive NSCLC 5 and correlate with a worse outcome 6. CDK4 overexpression in lung cancer may accelerate tumour progression and leads to an overall shorter survival time in lung cancer patients 7. In particular, *KRAS*-driven NSCLC is particularly dependent on CDK4 activity. Targeting this kinase in NSCLC has therefore been proposed as a therapeutic strategy in *KRAS*-mutant lung cancer that is resistant to conventional and targeted therapies 8.

A large number of CDK/cyclin inhibitors have been identified from natural substances, in high throughput screening assays, or through structure-guided approaches 9-13. The vast majority of these inhibitors are ATP-competitive, and there are currently more than 20 CDK inhibitors in clinical trials for anticancer therapeutics, including the FDA-approved ATP-competitive CDK4 inhibitors Palbociclib (PD-0332991), Abemaciclib (LY2835219), and Ribociclib (LEE011) 14-19. To date, these compounds suffer from an overall lack of selectivity and toxicity, and have had a limited success when used as single agents, but exhibit potent synergistic effects in combination with cytotoxic drugs such as cisplatin or paclitaxel. Moreover they induce emergence of resistant mutants 18, 20, highlighting the need to develop novel therapeutic approaches.

Targeting protein/protein interactions (PPIs) that are essential for enzyme function represents a particularly attractive alternative to ATP-competitive inhibitors. However, the modulation of PPIs with selectivity and potency represents a major challenge, given the highly conserved structural features of protein kinases. In this respect constrained peptides and peptidomimetics constitute privileged structures for the controlled display of key functional groups that interact with target surface hotspot residues, by virtue of their stable, well-defined and predictable conformation, and therefore constitute particularly attractive and tractable biomolecules for development of targeted therapeutics 21, 22*.* Especially, all-hydrocarbon stapled α-helical peptides have emerged as suitable pharmacological drug candidates with a large number of studies demonstrating their therapeutic potential 23-27. A leading example is the stapled peptide developed by Aileron Therapeutics, displaying an anti-tumor activity, and currently in phase I trials for solid tumor 28 and in phase II trials for lymphoma 29.

The primary interface between CDK and cyclin partners is mediated by the C helix of the CDK and the α5 helix of the cyclin partner 30, 31. Given its critical implication in assembly of an active CDK/cyclin complex, we previously showed that it constituted a molecular target and designed a peptide derived from the PSTAIRE helix of CDK2, which efficiently and specifically inhibited CDK2/Cyclin A 32. Targeting the primary interface between CDK4 and cyclin D could therefore constitute an attractive alternative to ATP-pocket binding compounds. In this study we have designed a stapled peptide derived from the C helix of CDK4, characterized by a stabilized helical conformation that binds cyclin D1 with high affinity, compared to a linear peptide derived from the same sequence. This stapled peptide penetrates readily into cultured cells, colocalizes with CDK4 and cyclin D1 and inhibits the ability of CDK4 to phosphorylate Rb. Moreover it inhibits lung cancer cell proliferation and efficiently prevents growth of orthotopic NSCLC tumours in mice when combined with the ATP-competitive inhibitor Abemaciclib.

## Materials and Methods

### Patients, tissue samples, and immunohistochemistry

Lung adenocarcinoma and squamous cell carcinoma samples were obtained form surgical rsections and retrieved from the Biological Resource Center of Grenoble University hospital (CRB) (authorized by Ministry of Higher Education and Research - Accreditation number AC 2017-2949- BRIF BB-0033-00069). All tumours were classified according to the 2015 WHO classification 1.

Immunohistostainings were performed on 3 µm formalin-fixed paraffin-embedded tissue sections on Benchmark Autostrainer (Ventana, Tucson, AZ). Sections were incubated with cyclin D1 rabbit mAb (clone SP4, ref # MA5-16356, Invitrogen, dilution 1:400), CDK4 rabbit mAb (clone, ref #12790, Cell Signaling Technology, dilution 1:400) and rabbit phospho-Rb (Ser807/811) mAb (clone D20B12, ref #8516, Cell Signaling Technology, dilution 1:200). Antigen retrieving was performed for cyclin D1 64 min in CC1 buffer (Ventana, Tucson, AZ), for pRB 60 min in CC1 buffer, and for CDK4 60 min in Novolink citrate buffer. The Ventana DAB detection kit (Ventana Medical Systems) was used according to the manufacturer's instructions. Omission of the primary antibody and/or incubation with same species and isotype IgG at the same concentration of the primary antibody served as negative controls. Pathologist blinded to clinico-pathological variables, mutation status and treatment response, independently evaluated the levels of expression using the percentage of positive tumour cells, with a cut off of positivity of >10% of stained cells.

### DNA extraction and sequencing

A 3 µm tissue section was stained with H&E (hematoxylin and eosin) and examined by light microscopy to assess the quality of all the samples and to determine areas containing more than 70% of tumour cells for microdissection before DNA extraction. DNA was isolated (QIAamp DNA mini kit, Qiagen, France) from 3 µm FFPE sections. The genotyping and mutation analyses were performed by using an accredited pyrosequencing method from Grenoble University Hospital clinical laboratory. The detailed methodology for tumor DNA sequencing has been previously described 33-35.

### Chemical synthesis and purification of peptides and stapled peptides

All reagents and solvents were obtained from commercial sources and used without further purification. Analytical HPLC analyses were run on an Agilent Technology 1220 Infinity LC equipped with a Chromolith Speed Rod RP-C18 185 Pm column (50 x 4.6 mm, 5 μm) with a gradient from 100% (H_2_O/TFA 0.1%) to 100% (CH_3_CN/TFA 0.1%) in 5 min; flow rate 4 mL/min; detection at 214 nm (conditions B). LC/MS analyses were recorded on a Quattro micro ESI triple quadrupole mass spectrometer (Micromass, Manchester, UK) equipped with a Chromolith Speed Rod RP-C18 185 Pm column (50 x 4.6 mm, 5 μm) and an Alliance HPLC System (Waters, Milford, USA); gradient from 100% (H_2_O/HCO_2_H 0.1%) to 100% (CH_3_CN/HCO_2_H 0.1%) in 3 min; flowrate 3 mL/min; UV detection at 214 nm. High-Resolution Mass Spectrometric analyses were performed with a time-of-flight (TOF) mass spectrometer fitted with an Electrospray Ionisation source (ESI) in positive ion mode.

Solid phase peptide synthesis were performed on an Amphispheres^®^ Rinkamide resin loaded at 0.38 mmol/g using Fmoc/*t*-Bu chemistry. First, resin was soaked in Dichloromethane (DCM) for 10 min and filtered. For each coupling reaction, 5 equivalents (eq.) of Fmoc-Amino Acid, 5 eq. of Hexafluorophosphate Azabenzotriazole Tetramethyl Uronium (HATU) and 10 eq. of N,N-Diisopropylethylamine (DIEA) were added to a fritted reaction vessel and stirred in N,N-dimethylformamide (DMF) (2 x 5 min at room temperature). For the coupling of olefinic amino acid, 3 eq. of Fmoc-(S)-pentenylalanine, 3 eq. of HATU and 5 eq. of DIEA were added to the reactor and stirred in DMF (2 x 30 min). Deprotection of the Fmoc group at the N-terminus was performed using a 20% piperidine/DMF solution (2 x 5 min at room temperature). After each coupling and deprotection step, resin was washed three times with DMF. After removal of the Fmoc group of the last amino acid, peptides P1, P2, P3, P4, MSI2, peptide control and P2*short* were acetylated at the N-terminus with a DIEA/Ac_2_O/DMF 1/1/8 vvv solution (2 x 10 min at room temperature).

The molecular stapling chemistry was first developed by Miller, Blackwell and Grubbs^36^-^38^, and further optimized by Verdine and Walensky group 23, 24, 39-43. Ring closing metathesis was directly performed on the solid support for peptides containing (S)-pentenylalanine residues in their sequence by using 0.4 eq. of Grubbs catalyst (first generation) in 1,2 dichloroethane, under inert atmosphere (stirred 2 x 2 hrs at r.t.), then resin was washed with 3 x DCM, 3x DMF, 3 x DMF.

Peptides were then cleaved from the resin with a TFA/TIS/H_2_O 95/2.5/2.5 vvv solution (2 × 90 min at r.t.). Resins were washed (1 x DCM, 1 x TFA, 1 x DCM) and filtrates were evaporated under reduced pressure. Compounds were precipitated by addition of 50 mL of diethyl ether and centrifuged (3000 rpm, 20 min). Crude peptides were then solubilized in acetonitrile/water 1/1 vv solution and purified by preparative RP-HPLC on a Waters system controller equipped with a C18 Waters Delta-Pack column (100 × 40 mm, 100 Å); flow rate 50 mL/min; UV detection at 214 nm using a Waters 486 Tunable Absorbance Detector and a linear gradient of A = H_2_O (0.1% TFA) and B = CH_3_CN (0.1% TFA). Peptides were recovered as TFA salts. Final compound purity was assessed by LC-MS analyses.

### Circular dichroism (CD) and Determination of Helical content

CD experiments were carried out using a Jasco J815 spectropolarimeter. Spectra were recorded with 100 μM of peptides dissolved in phosphate buffer 50 mM, pH 7, using a 1 mm pathlength CD cuvette at 20 °C, over a wavelength range of 190-260 nm. Continuous scanning mode was used, with a response of 1.0 s with 0.1 nm steps and a bandwidth of 2 nm. The signal to noise ratio was improved by acquiring each spectrum over an average of two scans. Baseline was corrected by subtracting the background from the sample spectrum.

Alpha helical content was determined using the following equation: % Helix = ([θ])_obs_ * 100)/(-39500 * (1-2.57/n), where ([θ])_obs_ is the mean residue ellipticity at 222 nm and n the number of peptide bonds.

### Fluorescent labelling of peptides

*Synthesis of Cy5.5-labelled P2short:* 1.8 mg (1.5 eq.) of Cyanine 5.5 NHS-ester was solubilized in 0.5 mL DMF (pH 12.0) and added to 3 mg (1 eq.) of P2shortA solubilized in 0.5 mL DMF (pH 12.0). The mixture was stirred for 2 h at room temperature and then purified by preparative RP-HPLC as described previously. DMF (pH 12.0) solution was prepared by adding 5 µL Et_3_N to 10 mL DMF.

*Synthesis of TAMRA-labelled P2short*: 1.035 mg (1.6 eq.) of TAMRA maleimide was solubilized in 0.1 mL DMF and added to 3 mg (1 eq.) of P2short solubilized in 0.7 mL Phosphate buffer (100 mM, pH 7). The mixture was stirred for 2 h at room temperature and then purified by preparative RP-HPLC as described previously.

*TP2-Rho-labelled peptides*: peptides were labelled on a unique cysteine residue with a five-fold molar excess of TP2-Rho-maleimide overnight, and then purified from free dye on NAP-5 columns (GE Healthcare), as described previously 44.

### Protein expression and purification of cyclins and cyclin-dependent kinases

Recombinant GST-CDK4 and GST-cyclin D1 were expressed in *E. coli* by IPTG induction and purified by FPLC chromatography as described previously 45.

Recombinant GST-cyclin A, GST-cyclin E and GST-cyclin Y were expressed in *E. coli* (BL21 DEA3) following induction with 1 mM IPTG overnight at 20 °C and purified by FPLC chromatography on a GSTrap HP 5 mL column (GE Healthcare) equilibrated in buffer A (50 mm Phosphate, pH 7.4, 150 mM NaCl). GST-tagged proteins were eluted with buffer A containing 50 mM glutathione (Euromedex), and then further injected onto a desalting column (GE Healthcare) equilibrated in buffer A to eliminate free glutathione. All proteins were expressed and purified freshly and their purety was verified by SDS-PAGE.

### Fluorescence Titration experiments

Fluorescence titration assays were performed in 96-well plates in a thermostated chamber (Clariostar spectrofluorimeter, BMG) at 30 °C in 200 μL phosphate buffer saline using 200 nM fluorescently-labelled peptides or proteins. The fluorescence emission intensity of TP2-Rho-labelled peptides was recorded at 614 nm following excitation at 510 nm. The fluorescence emission intensity of mant-ATP was measured at 356 nm following excitation at 448 nm. Data analysis was performed using the GraFit Software (Erathicus Ltd) and curve fitting was performed using a quadratic equation, as described previously 46. Experiments were performed in triplicate, and Kd values represent the average calculated for n = 3 to 5 experiments, together with standard deviation.

### Cell culture, extract preparation and Proliferation Assays

Non-small cell lung cancer A549, H358 and PC9 cell lines used in this study. Cell culture media, serum and antibiotics were purchased from Life Technologies. All cell lines were cultured in RPMI + Glutamax supplemented with 10% FCS, 100 units/mL penicillin (G sodium salt) and 100 µg/mL streptomycin at 37 °C in an atmosphere containing 5% CO_2_.

Cell extracts were prepared in PBS lysis buffer containing PBS (Sigma), pH 7.4, 150 mM NaCl, 0.2% NP40, 1 mM EDTA, 2 mM PMSF, CompleteTM protease inhibitors (Roche), and normalized following spectrophotometric dosage at 280 nm.

For cell proliferation/viability assays, cells were seeded in 96-well plates at 4,000 cells/well in 100 µL medium. 24 h later, cells were treated in quadruplicate with different concentrations of small molecule inhibitors or peptides (from 10 nM to 20 µM). Stock solutions of drugs were prepared in DMSO and freshly diluted in PBS to the desired concentration prior to use, then added onto unsynchronized cells cultured to subconfluency (60-70%), which were then further incubated for 24, 48 or 72 h. Cell proliferation was determined by crystal violet assay following treatment with drugs. Cells were washed with PBS, fixed with 3.7% formaldehyde for 10 min and then incubated with 0.1% crystal violet dye for 30 min. After rinsing, crystals were dissolved in 10% acetic acid and viability was determined by measuring absorbance at 595 nm.

### Western blotting

Cell extracts (30 µg) were separated on 12.5 % polyacrylamide gels, then electrotransferred onto PVDF membranes for Western blotting. Membranes were blocked with 5% BSA/TBS and then probed over night at 4 °C with antibodies. Anti-phospho-Rb (S807/811-D20B12), anti-CDK4 (D9G3E) and anti-actin (4967S) antibodies were purchased from Cell Signalling. Anti-cyclin D1 (sc-8396), anti-CDK4 (sc56361), anti-cyclin A (sc-751), anti-cyclin B (GNS1), and anti-cyclin E (sc-247) were purchased from Santa Cruz. Secondary anti-mouse (NXA931) and anti-rabbit (NA934) peroxidase conjugated antibodies were from Amersham.

### Pulldown experiments

The P2shortA stapled peptide was immobilized on activated CNBr resin (10-6 mole of peptide/0.3 g resin) (GE Healthcare) then saturated with PBS/1% BSA for 1 h at 4 °C prior to incubation with A549 cell extracts (60 µg/10 µL) for 1 h at 4 °C. The resin was then washed three times with PBS and boiled in Laemmli buffer for SDS-PAGE and Western blotting.

### Cellular Internalization, Indirect Immunofluorescence and Microscopy

TAMRA or Cyanine 5.5-labelled peptides were overlaid onto cultured cells grown to 50-60% confluency in DMEM 10% serum. Cells were then extensively washed with PBS and fixed with 3.7% formaldehyde/PBS for 10 min, washed twice and stained with Hoechst 33342 (Sigma) and either directly mounted onto glass slides in Mowiol for microscopic analysis or processed for indirect immunofluorescence. Following fixation, cells were incubated for 2 h in blocking buffer (4% BSA, 4% goat serum, 0.1% Triton) and indirect immunofluorescence was performed by incubating cells overnight at 4 °C with anti-CDK4 (sc-260, Santa Cruz) or anti-cyclin D1 (sc-717, Santa Cruz) diluted 1/500 in blocking buffer. Cells were washed four times with PBS for 5 min and then incubated for 1 h at room temperature with Alexa488-conjugated anti-rabbit antibody (R37116, Invitrogen) (diluted 1/500) followed by one wash in PBS for 5 min. Finally nuclei were stained with Hoechst 33342 (Sigma) diluted in H_2_O (1 μg/mL) for 5 min, and cells were washed in water and coverslips were mounted on glass slides in Mowiol. Fluorescent cells were observed with a Leica DM6000 microscope equipped with a CoolSnap HQ2 camera and piloted by MetaMorph software. Excitation band / dichroic / emission band filters for imaging fluorescent signals were as follows: Hoechst: 340-380/400/450-490 nm; TAMRA using the Rhodamine filters: Cyanine 5.5 using the Cy5 filters 590-650/660/662-737 nm; Alexa488-conjugated antibodies using the YFP filters.

### FACS experiments

Cells were trypsinized and fixed in 70% ice cold ethanol, washed in PBS and resuspended in PBS-1% BSA-50 mM citrate with 25 µg/mL RNase A for 15 min at 37 °C, supplemented with 25 µg/mL of propidium iodide for 30 min at room temperature. For each condition 20000 events were analysed by Gallios (Beckman Coulter) and data analysis was performed using FlowJo Software.

### In vivo biodistribution, pharmacokinetics, metabolism and tumour growth studies

#### *In vivo* tumour models

All animal experiments were performed in agreement with the European Economic Community guidelines and the “Principles of Laboratory Animal Care” (NIH publication N 86-23 revised 1985). Animal experiment studies were approved through institutional guidelines and by the European Community for the use of experimental animals (authorization to experiment APAFIS#5738-2016062010163562 v3).

##### Subcutaneous lung tumour models

Female NMRI nude mice (6-8 weeks old, Janvier, Le Genest-Saint Isle, France) were injected subcutaneously in the flank with 10×10^6^ H358 or A549 cells in 1X PBS. Tumour size was measured twice a week using a caliper, and the tumour volume was calculated as follows: length×(width)^2^×0.4.

##### Orthotopic lung tumour model

Luciferase-modified human H358 NSCLC cells (H358-Luc cells) were suspended in medium with 10 mM EDTA and 5 mg/mL Matrigel (BD Biosciences) at 5.10^6^ cells/50 µL. Six-week-old female NMRI nude mice (Janvier, Le Genest, Saint Isle, France) were anesthetized (isoflurane/air 4% for induction and 1.5% thereafter), and cells were inoculated in the lungs *via* the airways as previously described 47, 48. Non-invasive *in vivo* bioluminescence imaging (IVIS Kinetic, Perkin-Elmer) was used 10 min after the intraperitoneal injection of 10 mg/mL D-Luciferin (Promega, Charbonnières, France) to monitor the quality of the implantation and to follow orthotopic growth 47, 48. Five weeks after implantation, tumours were well developed and had a strong bioluminescent signal, but the mice did not present any detectable symptoms such as body weight loss or breathing difficulties.

#### Administration of fluorescent peptide *in vivo*

Cy5.5-labeled P2-short was administered to healthy mice, or mice bearing subcutaneous H358 or A549 tumours when they reached ~250 mm^3^, or to mice with orthotopic bioluminescent H358 lung tumours detected 5 weeks after implantation. Anesthetized mice (isoflurane/air 4% for induction and 1.5% thereafter) were injected intraperitoneally or intravenously *via* the tail vein with 200 µL of 8 µM (A549) or 10 µM (H358) Cy5.5-labeled P2-short in 5% glucose and 1% DMSO. Intrapulmonary administration of Cy5.5-labeled P2-short was performed using a nebulizing IA-1C Microspayer (Penn-Centur, Inc, PA, USA) connected to a FMJ-250-high-pressure syringe (Penn-Centur, Inc) containing 50 µL of 10 µM Cy5.5-labeled P2-short in 5% glucose and 1% DMSO, and as previously described 47, 48.

#### Pharmacokinetics on blood plasma samples

50 μL of blood were sampled from the tail vein of healthy mice before Cy5.5-labeled P2-short administration and at different times after, centrifuged (5 min at 8,000 g) and 10 µL of plasma were used for fluorescence imaging.

#### *In vivo* biodistribution

3D-fluorescence acquisitions were performed 24 h after intravenous or intrapulmonary administration, with the continuous-wave fluorescence-enhanced diffuse optical tomography system 48. fDOT consists of a 690 nm laser source, a CCD camera and a set of filters. The light source is a 35 mW compact laser diode (Power Technology, Little Rock, USA) equipped with a bandpass interference filter (685AF30OD6; Melles Griot, Albuquerque, NM, USA). Emitted fluorescence was filtered by 730/30 nm band-pass filter (RG9 OD5; Schott, Mainz, Germany) placed in front of a near infrared-sensitive CCD camera (Hamamatsu Photonics K.K., Japan) mounted with a f/15-mm objective (Schneider Kreutznach, Bad Kreuznach, Germany)^48^, ^49^.

2D-fluorescence images were acquired after administration using the Fluobeam 700^TM^ (Fluoptics, Grenoble, France) that excites fluorescence at 680 nm and detects the emitted light at wavelengths greater than 700 nm 50.

Mice were sacrificed at different times, and some organs, plasma and tumours were collected for *ex vivo* imaging using Optima Photon Imager optical imaging system (Biospace Lab). Semiquantitative data were obtained using the Wasabi^®^ software (Hamamastsu, Massy, France) by drawing regions of interest (ROIs) on the different organs and were expressed as the number of Relative Light Units per pixel per unit of exposure time and relative to the fluorescence signal in the skin.

#### Antitumour efficacy of P2-short in mice with orthotopic lung tumour

Five weeks after the inoculation of H358-Luc cells, when bioluminescent orthotopic tumours were detected, nude mice were randomly divided into 6 groups. Mice were treated with vehicle (control, n = 14), Abemaciclib (n = 10), or P2-short administered intravenously (n = 10) or intrapulmonary (n = 10), or treated with a combination of Abemaciclib and P2-short (intravenous administration of P2-short, n = 10; intrapulmonary administration of P2-short, n = 10). Mice were daily orally treated for three weeks with 10 mg/kg Abemaciclib. P2-short was either administered *via* the tail vein three times a week for three weeks under anesthesia (isoflurane/air 4% for induction and 1.5% thereafter) with 200 µL at 0.9 mg/kg, or administered intrapulmonary two times a week for three weeks under anesthesia with 50 µL at 0.45 mg/kg. Intrapulmonary administration of P2-short was performed using a catheter connected to a FMJ-250-high-pressure syringe (Penn-Centur, Inc) and introduced into the trachea of the animals using a dedicated laryngoscope. Mice were observed and weighed three times a week. Tumour growth was followed by *in vivo* bioluminescence imaging (IVIS Kinetic). After 3 weeks, mice were sacrificed, and blood samples were collected by cardiac puncture for biochemistry analysis (Vet16MScan II). Lungs were collected for immunohistochemistry analyses.

#### Immunohistochemistry

Lungs were frozen and sections of a 7 μm thickness were stained with hematoxylin and eosin (HE) or fixed for immunohistochemical staining.

Lung frozen sections were fixed with acetone and incubated with Ki67 antibody (1/500, Abcam Ab66155) for 2 h à room temperature, followed by incubation with goat anti-rabbit-HRP antibody (Dako) and DAB substrate (Dako). To determine proliferative index, total and Ki67 positive tumour epithelial cells were scored on two to eight fields per mouse in three or four mice per group, and reported as percentage of Ki67 positive cells. Results are reported as proliferative index in each field and each treatment group.

For p-RB staining, lung sections were fixed with 4% paraformaldehyde and heat mediated antigen retrieval with Tris/EDTA buffer, pH 9.0 was performed. Phospho-RB S807 antibody (1/4000, Abcam Ab184796) was incubated overnight at 4 °C, followed by incubations with ImmPRESS^TM^ HRP Polymer anti-rabbit IgG and ImmPACT^TM^ NovaRED^TM^ diluents (Vector Laboratories). Lung sections were imaged on an Olympus BX41 microscope. Total and p-RB positive tumour epithelial cells were scored on two to twenty-nine fields per mouse in three or four mice per group, and reported as percentage of p-RB positive cells in each field and each treatment group.

#### Statistical analyses

Statistical comparisons were made using Friedman test or Kruskall-Wallis test, with Dunn's multiple comparisons *posthocs* tests. *P* values ≤ 0.05 were considered statistically significant. All analyses were performed using the Graphpad Prism software.

## Results

### CDK4/cyclin D1 expression is correlated with the presence of *KRAS* mutation in lung cancer tumours

Since a synthetic lethal interaction has been reported between *KRAS* mutants and CDK4, we asked whether expression of cyclin D1 and CDK4 might be associated with the *KRAS* mutation in lung cancer patients. To this aim we first performed cyclin D1, CDK4 and pRb immunohistochemistry in a series of 215 NSCLC surgical resections. Clinical and molecular data are listed in **Table S1**.

**Figure 1:**
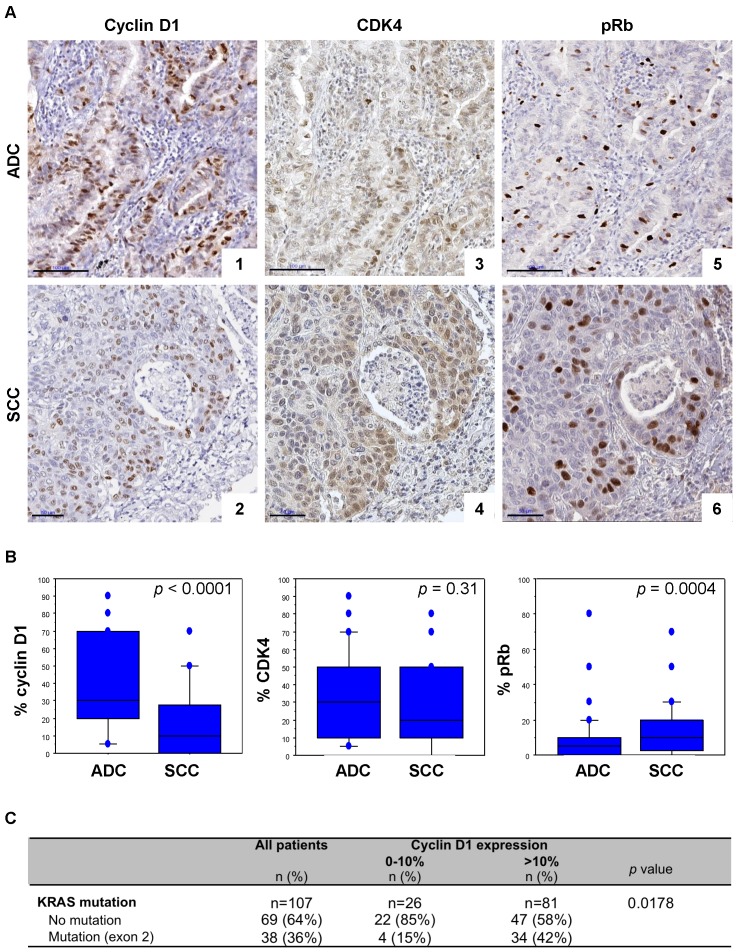
** Cyclin D1/CDK4 expression is correlated to the presence of *KRAS* mutation in lung cancer tumours. A.** pRb, CDK4 and cyclin D1 immunoperoxidase nuclear staining in lung adenocarcinoma (ADC, upper panels) and in squamous cell carcinoma (SCC, lower panels). CyclinD1 (panel 1, score of 70%; panel 2, score of 40%), CDK4 (panel 3, score of 50%; panel 4, score of 50%), and pRb (panel 5, score of 10%; panel 6, score of 20%) expression. Bars, 100 µm (ADC) and 50 µm (SCC). **B.** Distribution of cyclin D1, CDK4, and pRb staining (percentage of positive cells, Y-axis) according to the histological subtype of tumours. Statistical analysis was carried out using Mann-Whitney's *U*-test.** C.** Cyclin D1 expression according to *KRAS* mutation status of tumours. Statistical analysis was performed using Fisher's exact test. Missing data were excluded from the analysis.

High cyclin D1 expression was predominantly observed in ADC, whereas high pRb levels were more frequent in SCC (**Figure 1A, 1B** and**Table S1**). There was however no significant difference in CDK4 expression between ADC and SCC samples (**Figure 1A, 1B** and**Table S1**). A concomittant positive cyclin D1 and CDK4 expression, defined both by more than 10% of positive cells, was observed in 76% of the cases (**Table S2**), with a strong correlation (Spearman's correlation coefficient *r* = 0.420; *p* <0.0001). pRb expression was observed in 24% of the cyclin D1 positive cases and 30% of CDK4 positive cases (**Table S2**). A strong correlation between CDK4 and pRb (Spearman's correlation coefficient *r* = 0.239; *p* = 0.0005), and a trend between cyclin D1 and pRb expression in ADC (Spearman's correlation coefficient *r* = 0.235; *p* = 0.0155), but no significant correlation in SCC (Spearman's correlation coefficient *r* = 0.187;* p* = 0.064) were observed. Interestingly, a high level of cyclin D1 was significantly associated with *KRAS* exon 2 mutation: 89% of the *KRAS* mutant cases (all ADC) highly expressed cyclin D1 (**Figure 1C** and **Table S2**). Taken together these data highlight a correlation between cyclin D1/CDK4 expression and *KRAS* mutation in lung ADC, suggesting enhanced activation of CDK4/Cyclin D in *KRAS* mutant lung cancer.

### Design and synthesis of stapled peptides targeting the interface between CDK4 and cyclin D1

**Figure 2:**
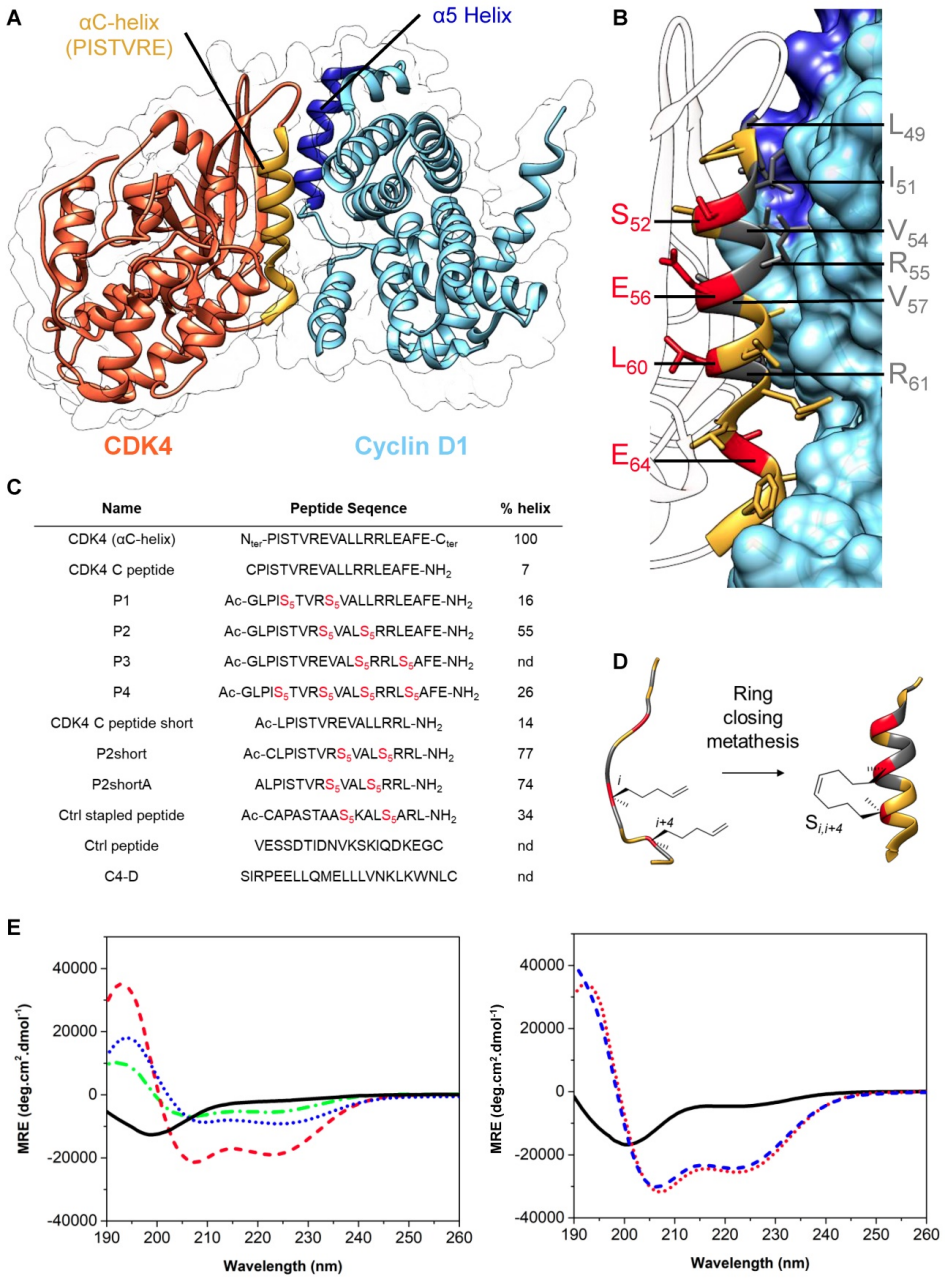
** Design & synthesis of foldamers targeting the interface between CDK4 and cyclin D1. A.** Crystal structure of human CDK4 in complex with a cyclin D1 (PDB: 2W9Z). **B.** Focus on the interaction between CDK4 C helix and cyclin D1; essential amino acids involved in this interaction are displayed in dark grey, residues in red were substituted by unnatural alkenyl amino acids to synthesize stapled peptides. **C.** Peptides used in this study, essentially derived from CDK4 C helix, from Cyclin D1 alpha 5 helix or controls; Ac corresponds to the N-terminal acetylation of the peptide and S_5_ corresponds to the (S)-pentenylalanine residue. Alpha helical content was determined using the following equation: % helix = ([θ])*_obs_* x 100)/(-39500 x(1-2.57/*n*), where [θ]*_obs_*is the mean residue ellipticity at 222 nm and *n* the number of peptide bonds.^1^ **D.** Schematic representation of stapled peptide synthesis by ring-closing metathesis. **E.** Overlay of far-UV CD spectra of the linear peptide CDK4 C peptide (*plain, black*) and three stapled peptides P1 (*dash-dot, green*), P2 (*dash, red*) and P4 (*dot, blue*). Typical α helix CD profiles display two negative bands at 208 nm and 222 nm and a positive band centered at 195 nm. **F.** Overlay of far‑UV CD spectra showing enhancement of alpha helical content between the linear peptide CDK4 C peptide short (*plain, black*) and the two stapled peptides, P2short (*dot, red*) and P2shortA (*dash, blue*).

The crystal structure of CDK4/cyclin D1 shows that the primary interaction between these partner proteins is mediated by the C helix in the N-terminal lobe of CDK4, also known as the PISTVRE helix, and the complementary α5-helix of cyclin D1 (**Figure 2A**) (51; PDB: 2W9Z). To inhibit this interaction, which is essential for CDK4 function, several peptides derived from CDK4 C helix were designed and synthesized as mimicks to compete with the CDK4/cyclin D1 interface. Specifically, we first designed a peptide derived from the C helix of CDK4 spanning residues G48 to L63 (G_48_L_49_P_50_I_51_S_52_T_53_V_54_R_55_E_56_V_57_A_58_L_59_L_60_R_61_R_62_L_63_) (**Figure 2**). This peptide is disordered in phosphate buffer, but become partially helical upon addition of 25% TFE (helicity of 46%), indicating its propensity to fold into an α-helix in the appropriate environment (**Figure S1A**).

To generate a stable and well-structured peptide derived from the CDK4 C helix, which would mimick its conformation in its native protein environment, and therefore constrain the peptide into an α-helix preserving the key residues required for interaction with Cyclin D1, we selected amino acids that were not involved in its interaction with cyclin D1 to introduce hydrocarbon staples. Close analysis of the crystal structure of CDK4/cyclin D1 complex 51 (**Figure 2A**) and implementation of the Drugscore PPI webserver 52 led us to identify L_49,_ I_51,_ V_54,_ R_55_V_57_ and R_61_ as critical residues positioned along the face of the C helix involved in the interaction with cyclin D1 (**Figure 2B** and **Figure S1B**). Residues S_52_, E_56_, L_60_ and E_64_, that lie on the opposite side of the C helix and point towards the core of CDK4 (**Figure 2B**), were therefore substituted by unnatural alkenyl amino acids at the position (i,i+4), followed by a ring closing metathesis reaction using ruthenium as catalyst to form the staple, to generate four stapled peptides spanning residues 48-67 (**Figure 2B, 2C, 2D**). P1, P2 and P3 comprise a unique staple, whereas P4 comprises two staples. Stapled peptide P3 was poorly soluble and was not investigated any further. Stapled peptides P1, P2 and P4 displayed typical α-helix CD profiles in phosphate buffer (two negative bands at 208 nm and 222 nm and a positive band centered at 195 nm), indicating that incorporation of staples in linear CDK4 C peptide induced α-helical conformation (**Figure 2E**).

Stapled peptide P2 displayed the strongest tendency to form a helix (55%) compared to P1 (16%) and P4 (26%) (**Figure 2C and 2E**). We further synthesized a shorter derivative of P2, named P2short, which lacks the four C-terminal residues (E_64_A_65_F_66_E_67_), and bears an N-terminal cysteine so as to enable site-specific fluorescent labelling for titration and cellular internalization experiments (**Figure 2C**), P2shortA, in which the cysteine was substituted by an alanine to prevent peptide dimerization through cysteine oxidation, and an unstapled analog, CDK4 Chelix short (**Figure 2C**). Both P2short and P2shortA displayed characteristic α-helical CD signatures, with a significant increase in helical content compared to P2, with 77% and 74% helical content, respectively, associated with deletion of the C-terminal tetrapeptide acidic patch in P2. As expected, substitution of the N-terminal cysteine by an alanine residue had no significant structural effect. CDK4 Chelix short peptide was disordered in solution (**Figure 2C, 2F**). Finally, to address the specificity of the interaction between CDK4-derived peptides and their target, as well as their anti-proliferative effect, two unrelated control peptides were synthesized: a stapled peptide of the same length as P2short (Ctrl stapled peptide) and an unstapled peptide (Ctrl peptide).

### Stapled peptides interact with cyclin D1 and sensitize CDK4 to ATP binding

In order to assess whether the helical conformation induced by staples improved the affinity of the peptides derived from the C helix of CDK4 for their intended target, cyclin D1, fluorescence titration experiments were performed with peptides labelled with TP2-Rho, a highly sensitive probe previously used to characterize peptide/protein interactions 44.

**Figure 3:**
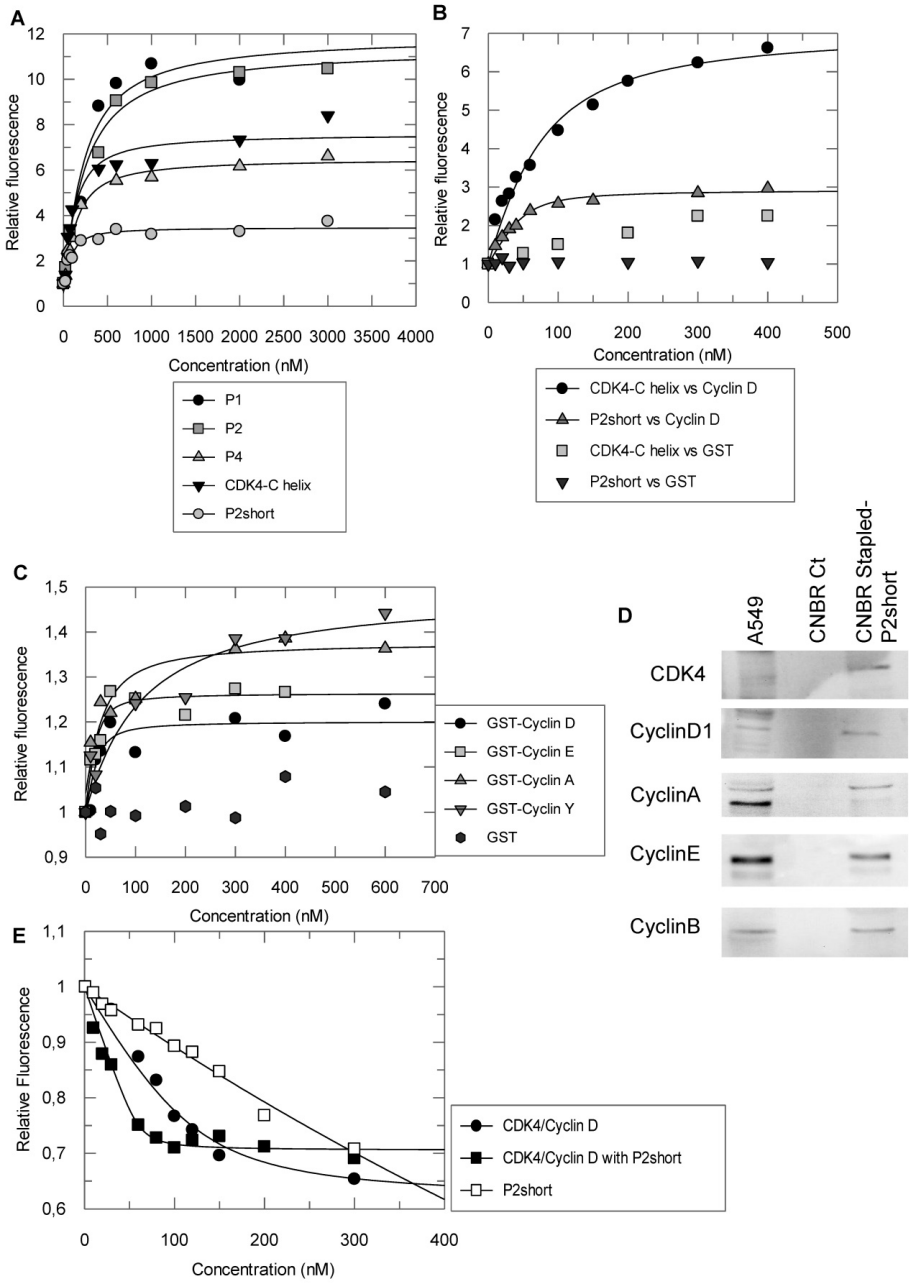
** Stapled peptides interact with cyclin D1 and sensitize CDK4 to ATP binding. A.** Fluorescence titration of 200 nM TP2-Rho labelled C4D peptide derived from the alpha 5 helix of cyclin D1 with CDK4 C helix peptide and stapled peptides P1, P2 and P4 **B.** Fluorescence titration of 200nM TP2-Rho labelled CDK4 C helix peptide or P2short peptide with GST-cyclin D1 and GST (left panel). An enlargement of the fit for titration of TP2-Rho P2short with GST-cyclin D1 is shown (right panel). **C.** Fluorescence titration of 200 nM TP2-Rho P2short with Cyclin A/E/D1/Y. **D.** Pulldown experiment: Western blotting of P2Short immobilized on CNBr Resin following incubation with A549 cell extracts.** E.** Fluorescence titration of 200 nM mant-ATP with CDK4/CyclinD1 or CDK4/cyclinD1/P2short. All experiments were performed n = 3 to 5 times. Representative curves are shown for each titration.

We first labelled a peptide derived from the alpha 5 helix of cyclin D1 (C4D peptide) to investigate its interactions with the peptides derived from CDK4 C helix. Titration of the TP2-Rho-labelled C4D peptide with unstapled CDK4 C helix peptide and stapled peptides P1, P2, P2short and P4 induced significant fluorescence enhancement in all cases and curve fitting and calculation of dissociation constants (Kd) yielded similar values for the CDK4 C helix peptide, P1 and P4 peptides (83 ± 45 nM, 140.5 ± 77 and 109 ± 29 nM, respectively) (**Figure 3A**). In comparison, P2 displayed 2-3 fold weaker affinity for C4D, with an average Kd of 245 +/- 84 nM, whereas P2short peptide exhibited 2-3 greater affinity for the C4D peptide with an average Kd of 56 +/- 19 nM, (**Figure 3A**). Taken together, these results indicate that stapling did not improve the affinity of peptides derived from the CDK4 C helix for the complementary peptide derived from α5 helix of cyclin D1, but that removal of the C-terminal acidic patch improved the affinity of the stapled P2short.

Conversely, the CDK4 C helix peptide and the P2short peptide were both labelled with TP2-Rho on their N-terminal cysteine. Fluorescence titration of the unstapled peptide with GST-cyclin D1 induced significant fluorescence enhancement (8 fold) indicating that it bound GST-cyclin D1, as expected, whereas GST alone had no significant incidence (**Figure 3B**). Interestingly titration of TP2-Rho-P2short peptide with GST-cyclin D1 induced only 3-fold fluorescence enhancement inferring that positioning of the probe conjugated to the constrained peptide upon binding to the recombinant cyclin most likely differed from that of the probe conjugated to the unstructured CDK4 C helix peptide. Curve fitting and calculation of dissociation constants revealed that the affinity of the P2short peptide for GST-cyclin D1 was four times greater than that of CDK4 C helix peptide, with average Kd values of 12 +/- 6 nM and 43 +/- 24 nM, respectively (**Figure 3B**). In contrast, TP2-Rho labelled control stapled peptide, did not induce any significant change in fluorescence upon titration with GST-cyclin D1.

Based on the affinity constants and physicochemical properties of the peptides (helicity and solubility), we selected the stapled peptide P2 and P2short for further characterization of their specificity for cyclin D1 and their inhibitory potential in NSCLC cells. Fluorescence titration of TP2-Rho-labelled P2short revealed very similar affinity for cyclins D1, A and E, but significantly lower affinity for the less conserved cyclin Y, with average Kd values of 12 +/- 6 nM, 25 ± 15 nM, 10 ± 7 nM, and 47 ± 40 nM, respectively **(Figure 3C).** In addition pulldown experiments performed with P2short peptide immobilized on CNBr Sepharose and incubated with A549 cell extracts confirmed that endogenous cyclins A, B, D1 and E were equally retained by P2short peptide (**Figure 3D**). Taken together these experiments show that P2short peptide, which is derived from a highly conserved region in CDKs, recognizes an equally highly conserved interface in cyclins involved in cell cycle progression.

Cyclin binding to a CDK induces a rotation of its N-terminal lobe, therefore aligning the ATP binding site with the catalytic cleft 31. In an attempt to determine whether P2short might bind cyclin D1 precomplexed to CDK4 and consequently impact the CDK4/cyclin D complex, we asked whether it might affect ATP binding. Interestingly, titration of the fluorescent analog mant-ATP with CDK4/cyclin D1 in the presence of P2short (2-fold excess) induced a 5-fold increase in the affinity of mant-ATP compared to its binding to the complex alone, with average Kd values of 5 +/- 2 nM and 27 +/- 13 nM, respectively (**Figure 3E**). Control titration of fluorescent mant-ATP with P2short peptide alone did not reveal any significant interaction. These results reveal that P2short can indeed bind cyclin D1 precomplexed to CDK4, and that this enables CDK4 to bind ATP more tightly, suggesting a possible conformational effect on CDK4.

### P2short penetrates readily into NSCLC cells, colocalizes with CDK4 and cyclin D1 and inhibits CDK4 activity and NSCLC cell proliferation

**Figure 4:**
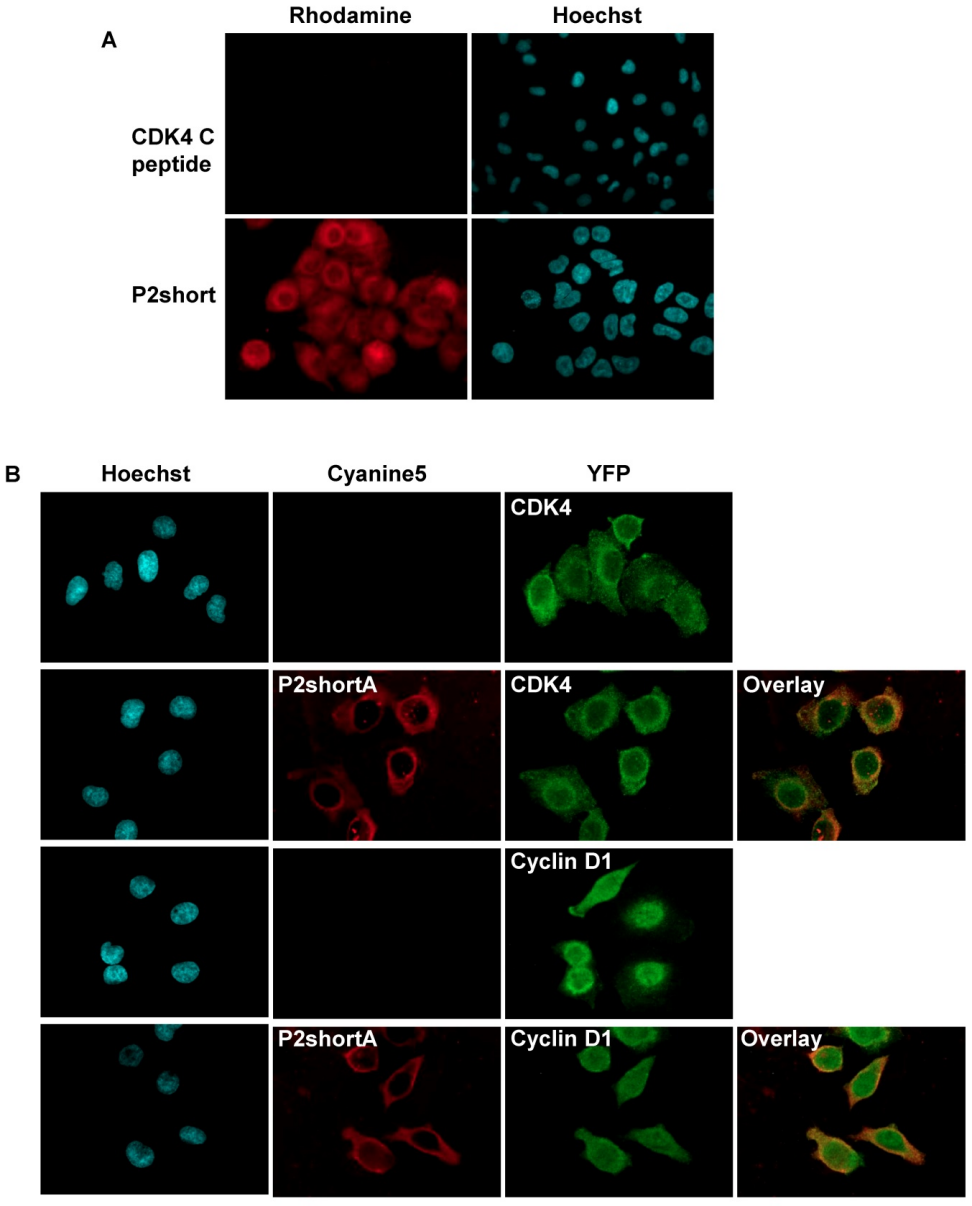
** P2short is internalized into A549 cells and colocalizes with CDK4 and cyclin D1. A.** A549 cells were cultured with 1 µM TAMRA-labelled CDK4 Chelix peptide or P2short peptide for 1 h. Representative confocal microscopy images of peptides. In red: TAMRA-labelled peptide fluorescence associated with peptide internalization; in blue: Hoechst-stained nuclei. Magnification 63X. **B.** Representative images of peptides, and CDK4 and Cyclin D1 immunodetection. The overlay of internalized Cy5.5-labelled P2shortA with CDK4 or Cyclin D1, respectively reveals partial colocalization in the cytoplasm. In red: Cy5.5-labelled P2shortA peptide fluorescence; in green: CDK4 or Cyclin D1 immunofluorescence; in blue: Hoechst-stained nuclei. Magnification 63X.

The ability of TAMRA-labelled CDK4 C helix and P2short peptides to enter A549 cells was investigated by fluorescence microscopy. As expected for most peptides, the CDK4 C helix peptide was unable to cross the cell membrane **(Figure 4A)**. In contrast, P2short internalized into A549 cells and exhibited very intense cytoplasmic fluorescence emission within 1 h **(Figure 4A)**. Indirect immunofluorescence experiments revealed that P2short partially colocalized with CDK4 and cyclin D1 in the cytoplasm **(Figure 4B)**. Moreover comparison of CDK4 and cyclin D1 localization in untreated cells and cells treated with P2short did not reveal any differences, indicating that P2short did not directly affect the subcellular localization of its targets.

**Figure 5:**
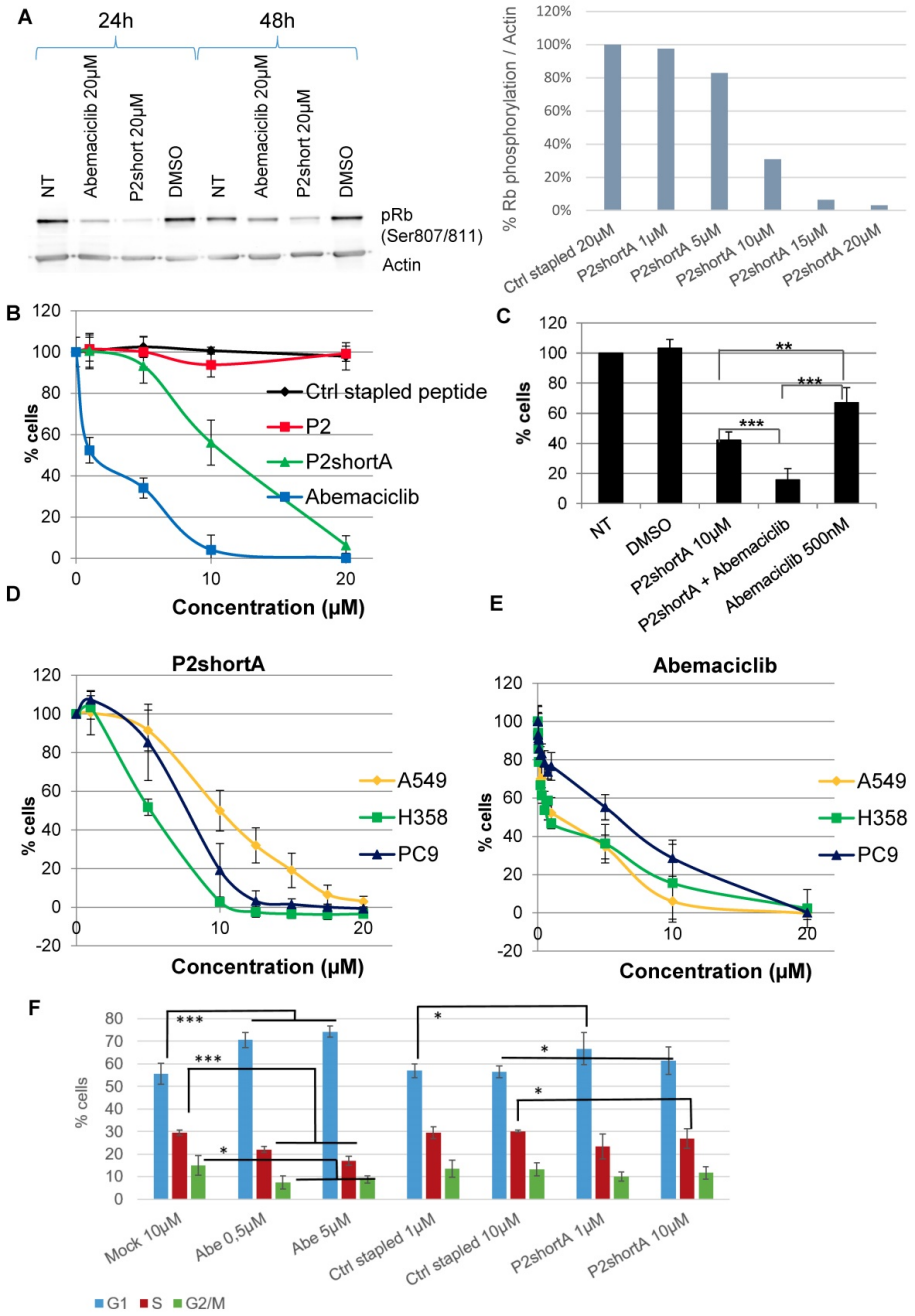
** P2short inhibits CDK4 activity and NSCLC proliferation but does not block cell cycle arrest. A.** p107Rb phosphorylation (Ser807/811) was determined by Western blotting of A549 cell extracts prepared following treatment with Abemaciclib or P2short peptide. Actin levels were detected to control constant protein loading. Left panel: Rb phosphorylation after 24 and 48 h, following treatment with 20 µM Abemaciclib or P2short peptide. Right panel: quantification of the relative Rb phosphorylation/actin levels in after 24 h following treatment with 20 µM Ctrl stapled peptide or different µM concentrations of P2short peptide. **B-E.** *Proliferation assays.* **B.** A549 cells were treated with different concentrations of Ctrl stapled peptide, P2, P2shortA and Abemaciclib. **C.** A549 cells were treated with combined administrations of P2shortA peptide (10 µM) and Abemaciclib (500 nM) Bars are averages of three independent experiments (n=4/experiment) ± SD. Means were compared with t test, * p<0.05,** p<0.02, *** p<0.01. **D-E.** PC9, H358 and A549 cells were treated with different concentrations of P2shortA (**D**) or Abemaciclib (**E**). Results are presented as average of at least two independent experiments (n = 4/experiment) ± standard deviation. **F.** Flow cytometry of A549 cells treated with DMSO, 0.5 or 5 µM Abemaciclib (Abe) and/or 1 or 10 µM Ctrl stapled or P2shortA peptide for 24 h. Means were compared with *t* test, * p<0.2,** p<0.1, *** p<0.05.

We therefore investigated the effect of P2short peptide on CDK4 activity in NSCLC cells, by addressing its ability to inhibit phosphorylation of p107Rb, the main substrate of CDK4. P2short effectively inhibited phosphorylation of p107 Rb in A549 cells, and to a greater extent than the ATP-competitive inhibitor of CDK4 Abemaciclib, after both 24 h and 48 h, and in a dose-dependent fashion (**Figure 5A**). The inhibitory efficacy of P2short was further assessed in NSCLC proliferation assays, and compared to the ATP-competitive CDK4/CDK6 inhibitor Abemaciclib.

To avoid dimerization in solution through disulfide bond formation and any possible inconsistencies that could arise over time in cellular studies, we resorted to use the analog P2shortA. Comparison of P2short and P2shortA in proliferation assays revealed very similar results, inducing a dose-dependent inhibition of A549 cell proliferation with a significant effect at 5-10 μM (**Figure 5B, Figure S2A and S2B**). Abemaciclib induced more cytotoxicity than P2shortA (**Figure 5B and Figure S2D**). Calculation of IC_50_ values for P2shortA and Abemaciclib in A549 cells yielded 10.81 ± 0.26 µM and 2.72 ± 2.21 µM, respectively **(Figure S2C** and** S2E)**. In contrast the longer P2 stapled peptide had no inhibitory effect whatsoever, nor did the Ctrl stapled peptide **(Figure 5B)**. Interestingly, coadministration of 10 μM P2short with a sub-IC_50_ concentration of Abemaciclib (500 nM) revealed a synergistic effect between these two compounds **(Figure 5C)**, with more than 80% inhibition of A549 proliferation, in agreement with in vitro studies described above in which ATP analog binding to CDK4/cyclin D1 was increased by P2short (**Figure 3E**). Finally, comparative proliferation inhibition studies performed on several NSCLC cell lines with mutant *KRAS* (A549 and H358) or wild-type *KRAS* (PC9) revealed that P2short was more potent than Abemaciclib in all cases **(Figure 5D, 5E)**.

Since CDK4 is essentially active during the G_1_ phase of the cell cycle, and its inhibition by Abemaciclib is reported to promote accumulation of cells in G_1_, we analysed the effect of P2short on the cell cycle of A549 cells. As expected, at both 0.5 µM and 5 µM Abemaciclib significantly increased the proportion of cells in G_1_ after 24 h treatment, whereas Ctrl stapled peptide had no effect even at 10 µM. In comparison, cells treated with P2short only exhibited a slight trend to accumulate in G1 after 24 h following treatment with either 1 or 10 µM P2short, compared to mock-treated cells, (**Figure 5F**).

### P2short biodistribution and pharmacokinetics profile in mice

The behaviour of Cy5.5-labeled P2shortA was first assessed in mice with human NSCLC tumour following intravenous, intraperitoneal, or intrapulmonary administration. P2shortA administered intraperitoneally was mainly detected in the abdomen of mice with A549 subcutaneous tumour, suggesting the non-specific binding of the peptide in the peritoneal cavity (**Figure S3A and S3C**). In contrast, P2shortA administered intravenously circulated at 5 h and was observed in the liver and kidneys after 24 h in mice with A549 or H358 subcutaneous tumour, suggesting hepatic and renal elimination of the peptide (**Figure S3B and S3C**). Based on these results, intraperitoneal injection was not continued, and the biodistribution of Cy5.5-labeled P2shortA following intravenous administration was compared to intrapulmonary administration using orthotopic mouse models of human NSCLC and healthy mice.

**Figure 6:**
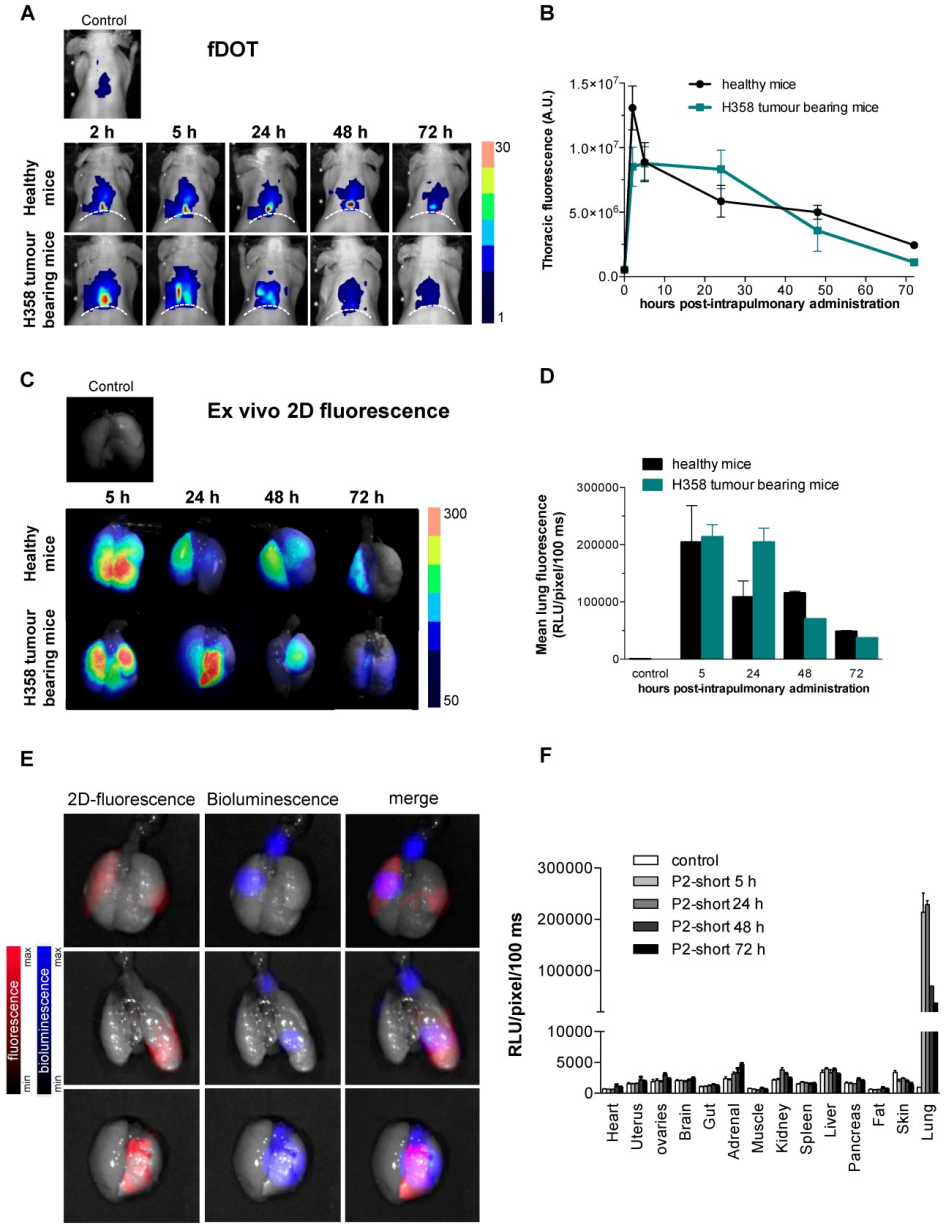
** Cy5.5-P2shortA accumulates in the lungs after intrapulmonary administration in mice with orthotopic H358 tumour.** 10 µM Cy5.5-P2shortA were administered intrapulmonary (50 µL) in healthy and orthotopic H358 tumour-bearing mice. **A.** 3D-fluorescence tomography (fDOT) imaging was performed at the indicated time after administration of Cy5.5-P2shortA. Dotted line: position of diaphragm. **B.** Volumes of interest are defined on the thoracic region to semi-quantify the total amount of photons. The results are expressed as the mean ± SEM in healthy (5 h, n ≥ 5; 24 h, n ≥ 3) or H358 tumour-bearing mice (5 h, n ≥ 6; 24 h, n ≥ 2). **C.** Fluorescent images were performed on isolated lungs at the indicated time after Cy5.5-P2shortA administration.** D.** Regions of interest (ROIs) are defined on the extracted lungs to semi-quantify the amount of photons detected per pixel after a 100 ms exposure. The results are expressed as the mean ± SEM in healthy (n = 4), or H358 tumour-bearing lungs (n ≥ 3), or non-injected healthy mouse (control). **E.** Bioluminescence, and 2D-fluorescence imaging were performed on isolated 24 h after intrapulmonary administration of Cy5.5-P2shortA. Bioluminescent signal showed H358-Luc tumour cells in lungs (in blue); 2D-fluorescent signal showed Cy5.5-P2shortA location in the lungs (in red); fluorescent and bioluminescent signals were merge. Cy5.5-P2shortA signal and H358 tumours were partially colocalized in lungs 24 h after intrapulmonary administration of Cy5.5-P2shortA. **F.** ROIs are defined on the organs to semi-quantify the amount of photons detected per pixel after a 100 ms exposure. The results are expressed as the mean ± SEM in healthy (n = 4), or H358 tumour-bearing lungs (n ≥ 3), or non-injected healthy mouse (control).

Cy5.5-labeled P2shortA fluorescence was detected in the thoracic region of healthy mice or mice with H358 orthotopic lung tumour, by *in vivo* 3D-fluorescence tomography (**Figure 6A**). Thoracic P2shortA fluorescence remained stable over 24 h after intrapulmonary administration and slowly decreased until 72 h (**Figure 6A and 6B**). No significant difference was observed between healthy and mice with H358 lung tumours. The fluorescence of the lungs e*x vivo* was elevated, remained constant over the next 24-48 h, and was similar in mice bearing tumours or not (**Figure 6C and 6D**). Moreover P2shortA fluorescence signal co-localized with the bioluminescent signal in lung tumours, inferring that intrapulmonary P2shortA indeed reached the lung tumours (**Figure 6E**). No significant fluorescence signal was observed in organs other than lungs (**Figure 6F**), nor in plasma (**Figure S4A**), thus confirming the strong retention of the P2shortA in the airways.

In contrast, P2shortA administered intravenously rapidly increased in the thorax at 2 h and then decreased over 24 h (**Figure S4B and S4C**). The fluorescence of the lungs e*x vivo* showed little P2shortA signal 5 h after intravenous injection, ten times lower than after intrapulmonary administration, and no signal at 24 h (**Figure S4D and S4E**). In addition, no colocalization was observed between the weak P2shortA fluorescence signal and the bioluminescent signal in the lung tumours (**Figure S4F**). P2shortA fluorescence was detected in several organs 5 h following intravenous injection, indicating that the peptide circulated largely in the different organs without any specific targeting (**Figure S4G**). This was further evidenced by the detection of P2shortA in plasma samples of healthy mice at different times following intravenous injection (**Figure S4A**). P2shortA was also detected in the liver and kidneys at 24 h (**Figure S4G**).

Taken together, these results reveal that P2shortA remains in the bloodstream several hours after intravenous injection without targeting lung tumours, before its hepatic and renal elimination. In contrast, P2shortA accumulates and remains in the lungs for several days after intrapulmonary administration, where it partially co-localizes with the lung tumours.

### *In vivo* administration of P2shortA reduces H358 orthotopic lung tumour growth when co-administered with Abemaciclib

**Figure 7:**
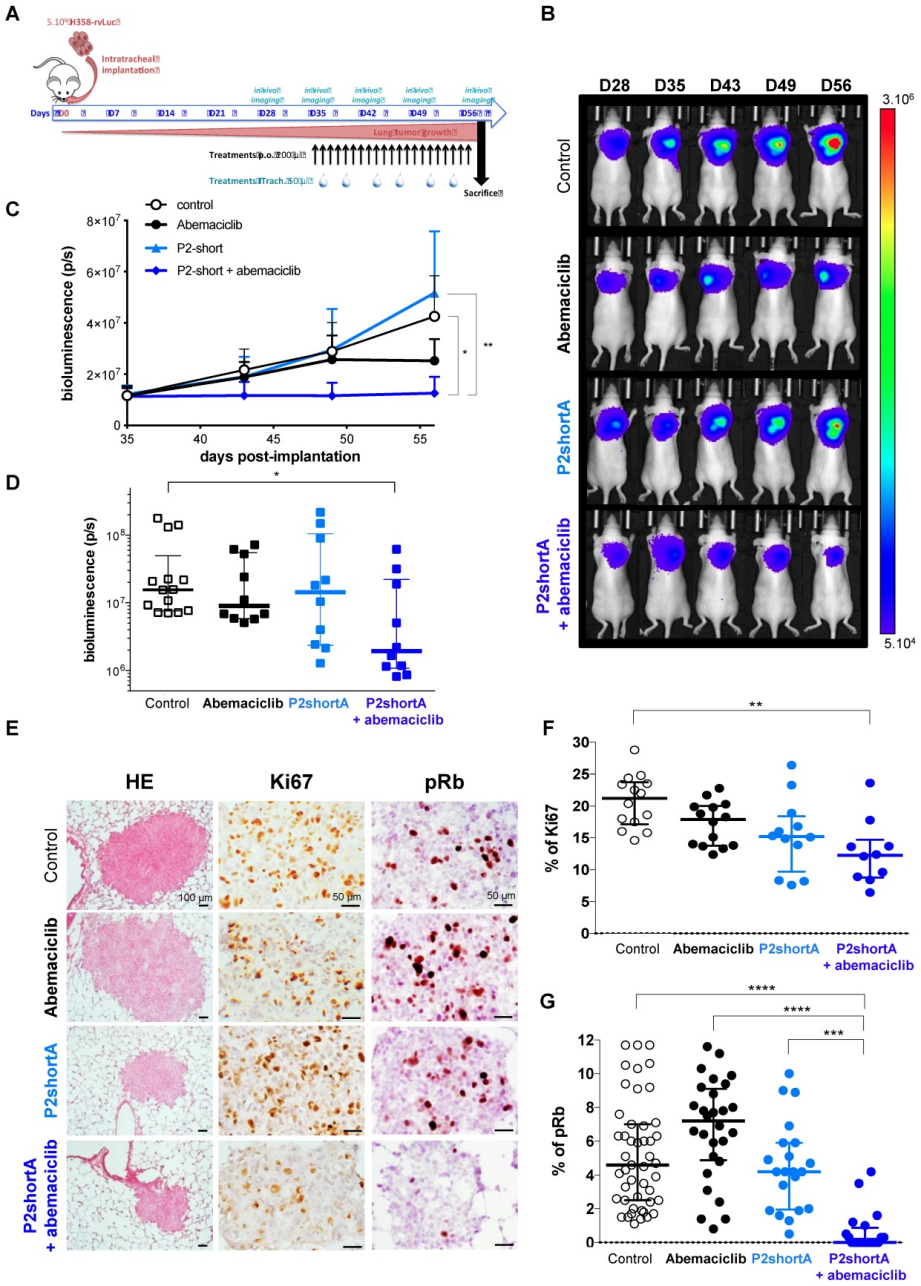
** P2shortA inhibits orthotopic H358 lung tumour growth when co-administered with Abemaciclib. A.** Schematic representation of H358 orthotopic tumours growth and of the treatments plan. The mice were inoculated with H358-rvLuc cells and randomized after 5 weeks into 3 groups of 10 mice and one control group of 14 mice. Vehicle or abemaciclib 10 mg/kg were administered *per os* every days for 3 weeks. 0.45 mg/kg P2shortA was administered intrapulmonary 2 times a week for 3 weeks. Thoracic bioluminescence imaging was performed once a week to follow tumour growth. **B.** Overtime thoracic bioluminescence images of H358-Luc tumours. One representative mouse (dorsal view) per group is shown. **C.** P2shortA and Abemaciclib combination inhibited the growth of orthotopic H358 tumours. The results are expressed as the mean ± SEM (control group, n = 14; treated groups, n = 10). Friedman test with Dunn's multiple comparisons *posthoc* tests (*p* = 0.0026); *, treatment combination group compared to control group; **, treatment combination group compared to P2shortA group. **D.** Thoracic bioluminescence level in each mouse at day 56 showed reduced level in P2shortA and Abemaciclib combination group. Bars, medians with interquartiles. Kruskall-Wallis test with Dunn's multiple comparisons *posthoc* tests; *, treatment combination group compared to control group. **E.** Histological (HE) and immunohistochemistry for Ki67 and phosphorylated-Rb (pRb) on lung tumour sections. Scale bars, 100 µm (HE) and 50 µm (Ki67 and pRb). **F.** Ki67-positive cells were quantified on different fields (2-7 fields) in three mice per group, and reported as percentage of Ki67 positive cancer cells. Bars, medians with interquartiles. Kruskall-Wallis test with Dunn's multiple comparisons *posthoc* tests (*p* = 0.0042); **, treatment combination group compared to control group. **G.** pRb-positive cells were quantified on different fields (2-29) in three or four mice per group, and reported as percentage of pRb positive cancer cells. Bars, medians with interquartiles. Kruskall-Wallis test with Dunn's multiple comparisons *posthoc* tests (*p* < 0.0001); ***, treatment combination group compared to P2shortA group; ****, treatment combination group compared to control or Abemaciclib groups.

Based on the results of the biodistribution and cell proliferation studies, we performed therapeutic studies to address the *in vivo* cooperation of P2shortA and Abemaciclib using H358 orthotopic lung tumours in mice. Five weeks after the H358 cells were inoculated into the lungs and the tumours established, the mice were randomly divided into different groups and treated for three weeks with vehicle (control group, n=14), Abemaciclib (daily oral treatment, 10 mg/kg, n=10), P2shortA (administered intrapulmonary 2 times a week, 0.45 mg/kg, n=10), or a combination of Abemaciclib and P2shortA (n=10) (**Figure 7A**).

The concentration of Abemaciclib was chosen based on a preliminary experiment, so as to have a sub-therapeutic effect on the H358 orthotopic lung tumour model. Bioluminescence follow-up of the tumours indicated that the low concentration of Abemaciclib slightly reduced tumour growth, whereas P2shortA had no effect (**Figure 7B, 7C and 7D**). Interestingly, the combination of Abemaciclib and P2shortA significantly inhibited tumour growth as compared to the control or P2shortA-treated groups (**Figure 7B-D**). In contrast, P2shortA administered intravenously (0.9 mg/kg, 3 times a week, n=10) alone or in combination with Abemaciclib showed the same modest reduction of tumour growth, similar to Abemaciclib alone (**Figure S5A-D**).

All treatments were well tolerated. No significant weight loss was observed in the H358 tumour-bearing mice (**Figure S6A**), and no significant change in serum biochemical values was observed irrespective of treatments (**Figure S6B and S6C**). Taken together these results reveal that intrapulmonary P2shortA restrict tumour growth over time when combined with Abemaciclib, whereas intravenous P2shortA failed to have any therapeutic effect probably because it was not retained long enough in the tumour at the concentration used.

Analysis of tumours by Hematoxylin and eosin (HE) staining did not reveal any lung damage following intrapulmonary P2shortA administration, but pulmonary toxicity will have to be formally assessed to confirm the safety of P2shortA (**Figure 7E**). Tumour sections stained with Ki67 and pRb revealed a significant reduction of both Ki67-positive cells and pRb-positive cells in tumours following intrapulmonary administration of P2shortA combined with Abemaciclib, indicative of a reduced proliferative index and reduced CDK4 activity, respectively (**Figure 7E-G**). In contrast intrapulmonary administration of P2shortA or Abemaciclib alone did not reduce Ki67 or pRb levels, indicating that, at least in this experimental design, and at the concentrations of treatments chosen, tumour growth remain unaffected (**Figure 7E-G**). Likewise, intravenous administration of P2shortA either alone, or in combination with Abemaciclib did not promote significant reduction of Ki67-positive cells (**Figure S5E and F**). In contrast, intravenous administration of P2shortA with Abemaciclib led to a much broader distribution of pRb-positive cells (**Figure S5E and G**). Taken together these results confirm that intrapulmonary administration of P2shortA inhibits proliferation when it is combined with Abemaciclib in H358 orthotopic lung tumours *in vivo* by effectively inhibiting Rb phosphorylation, due to dual targeting of CDK4/cyclin D1.

## Discussion

CDK4/Cyclin D kinase constitutes an established biomarker in lung cancer, hyperactivity of which is associated with mutations in the p16^INK4a^-cyclin D-CDK4/6-pRb pathway 9, 11, 14, 53, 54. In agreement with other studies 5, 7, we found high levels of cyclin D1 and CDK4 in invasive NSCLC samples. Our results showing the direct association between expression of cyclin D1, CDK4, and high pRb levels, strongly suggest activation of cyclin D1/CDK4 pathway in lung adenocarcinoma. Furthermore, the strong correlation of cyclin D1 overexpression with mutated *KRAS* that we report in human NSCLC tumours, underscores the attractivity of CDK4/cyclin D as a pharmacological target for development of anticancer therapeutics, in particular in *KRAS*-mutant lung cancer, which has been described to be particularly dependent on CDK4 8.

Although most FDA-approved therapeutics targeting protein kinases are ATP-competitive small molecules 19, recently-approved CDK4/6 inhibitors exhibit limited potency when used as single agents, and require combined therapies to enhance their efficacy including letrozole or fulvestrant in breast cancer, cisplatin, paclitaxel, the proteasome inhibitor bortezomib or dexamethasone in multiple myeloma 15, 55-57 Moreover, the lack of selectivity of current CDK4/6 inhibitors induces several adverse secondary effects, including life-threatening blood clots in the lung arteries leading to pulmonary embolism 17, 58, and their mechanism of action ultimately leads to emergence of resistance 18, 20. Hence the limitations associated with their mechanism of action call for alternative strategies to restrict kinase hyperactivity in pathological conditions.

Compounds that target essential protein/protein interfaces constitute attractive and promising therapeutic alternatives to ATP-pocket binding compounds 32, 59. Here we describe the characterization of stapled peptides derived from the α-helix of CDK4 involved in the primary interaction with cyclin D1, designed to inhibit CDK4/cyclin D. These stapled peptides fold into an α-helix in solution in the absence of cyclin D1, and interact with cyclin D1 with high affinity compared to the unstapled peptides. Indeed, as usually observed for peptide sequences extracted from their proteic environment, linear peptides (CDK4 C and CDK4C short) derived from the C helix of CDK4 did not fold into an α-helix spontaneously. In contrast constraining these peptide sequences with hydrocarbon staples induced an α-helical conformation. This structural property has been shown to limit the susceptibility of linear peptides to protease degradation, to enhance their potential to interact with their target and to cross cell membranes 39, 40. Peptide stapling is nowadays considered as the “gold standard” to turn a peptide into a biologically active helix 60. The molecular stapling strategy we used in this study and previously described by Verdine et al. 43 is based on incorporation of modified amino acids (α-methyl, α-alkenylglycine) into strategic positions of a peptide sequence to constrain and induce stable α-helical secondary structures and expose critical amino acids involved in an interaction. Our studies revealed that one staple was largely sufficient to induce helical folding, but that the position of the staple itself was crucial to increase helical contant (P2 versus P1). Moreover, deletion of the C-terminal acidic patch of P2, which is not fully helicoidal in the crystal structure of CDK4/cyclin D1, significantly increased the α-helical content of the stapled peptide P2short (from 55 to 77%). It is noteworthy that the helical content correlated with the affinity of the peptide for cyclin D1.

The P2short peptide was capable of penetrating into cultured cells rapidly and efficiently, in contrast to the native unstructured peptide. In addition to its helical content, internalization of P2short was probably enhanced by its physicochemical properties, since deletion of the acidic patch also yields a net positive charge (+3 and +4 for P2short and P2shortA, respectively), which has previously been reported to be optimal for cellular internalization of stapled peptides 39. Our data also showed that P2short displayed higher affinity for cyclin D1 than P2 and that the latter was unable to inhibit cell proliferation. Overall our results support that both stabilization of the α-helix derived from CDK4 through introduction of a staple and the net positive net charge of P2short peptide contributed to its high biological efficiency.

Although the P2short peptide was derived from CDK4 and interacted with cyclin D1 with high affinity, we also found it bound other cyclins in vitro and in cell extracts. This is not surprising since the amino acids involved in the α5-helix that interacts with the C helix of CDKs are highly conserved. This lack of selectivity for cyclin D1 was also evidenced by the flow cytometry experiments, which did not reveal any accumulation of cells treated with P2shortA in G_1_, as would be expected from a CDK4/cyclin D1 inhibitor, and observed for Abemaciclib. Furthermore fluorescence microscopy and indirect immunofluorescence studies showed that P2short partially colocalized with CDK4 and cyclin D1, but did not induce subcellular relocalization of these proteins. However, P2short indeed induced a decrease in Rb phosphorylation to a similar extent as Abemaciclib, and also inhibited proliferation of NSCLC cell lines. Taken together, our results indicate that P2short indeed affects the ability of CDK4/cyclin D1 to phosphorylate its substrate. However, since P2short binds several cyclins, it might also affect the kinase activities of several CDKs, thereby explaining the overall inhibition of cell proliferation, but the lack of an observable accumulation of cells in a specific cell cycle phase.

Despite an antiproliferative effect on several NSCLC cell lines, intravenous injection of P2short failed to inhibit lung tumour growth in mice, most likely because the concentration retained at the tumour site was too low. In contrast, direct administration of P2short into the airways was associated with its strong accumulation in the tumours, as well as throughout the healthy parts of the lungs, but not in any other organs. Intrapulmonary administration of antitumoral agents can enhance their concentration at the targeted lung cancer site, and has already been successfully reported for lung cancer treatment 47, 48, 61, 62. However, direct intrapulmonary administration of P2short alone was insufficient to inhibit lung tumour growth at the concentration used, which was very low. When combined with the ATP-competitive inhibitor Abemaciclib both in cultured cells and *in vivo*, it did however completely inhibit growth of orthotopic NSCLC tumours in mice. This original combined effect between a therapeutic peptide targeting an essential PPI and a SMKI (small molecule kinase inhibitor) has a very strong potential for reducing the doses required to treat lung cancer. Indeed, it has been shown that drug combinations achieve favorable outcomes such as enhanced efficacy, decreased dosage, reduced or delayed development of drug resistance indicative of the beneficial combination of drugs with different targets or mechanisms of action 63, 64. In addition, stapled peptides have emerged as attractive pharmacological drug candidates 23, 25-29. This mechanism will require further investigation, but a reasonable explanation would be that Abemaciclib treatment promotes accumulation of cells in G_1_, where the availability of monomeric CDK4 and cyclin D1 are much greater than in other phases of the cell cycle, since CDK4 is degraded during mitosis and resynthesized in G_1_ 65. The window of opportunity for P2short to bind cyclin D1 would therefore be potentially greater in G_1_ than during the other phases of the cell cycle where the CDK4/cyclin D1 complex is already assembled. Our in vitro studies have shown that P2short enhances the affinity of recombinant CDK4/cyclin D kinase for ATP, suggesting that P2short might in turn increase Abemaciclib binding and efficacy. The interaction between P2short and cyclin D1 might not preclude CDK4/Cyclin complex formation, since we did not observe either subcellular delocalization of either partner upon treatment with P2short, nor disruption of the recombinant complex *in vitro* (data not shown), but might instead enact on the kinase complex conformation, thereby promoting increased binding of Abemaciclib in the ATP pocket, in line with our experimental results with the fluorescent ATP analog. Hence by treating cells with an ATP-competitive inhibitor that accumulates cells in G1, this would improve accessibility and targeting of P2short, which would in turn sensitize CDK4 to Abemaciclib.

In conclusion, the strategy described here consisting in targeting an essential protein/protein interaction, mediated by two α-helices, with a stapled peptide derived from one of these interfaces, for which we have provided a convincing proof-of-concept by successfully targeting CDK4/Cyclin D *in vitro* and *in vivo*, constitutes a promising therapeutic strategy to target the hyperactivity of CDK4 in lung cancer with mutant *KRAS*. In addition, it is very unlikely that an essential PPI will undergo mutations leading to emergence of resistance, in contrast to the ATP pocket of protein kinases as classically observed in patients following treatment with ATP-competitive drugs such as Erlotinib or Gefinitib. Our stapled P2short peptide thus constitutes an attractive means of sensitizing CDK4 to ATP-competitive therapeutics for the treatment of KRAS-mutant lung cancer patients.

## Abbreviations

ADC: adenocarcinoma
CDK: cyclin dependent kinase
HE: hematoxylin and eosin
KRAS: Kirsten rat sarcoma viral oncogene homolog
NSCLC: non-small cell lung carcinoma
PPI: protein/protein interactions
pRb: phosphorylated Rb
SCC: squamous cell carcinoma
