# Supplementary material for: Stapled peptide targeting the CDK4/Cyclin D interface combined with Abemaciclib inhibits KRAS mutant lung cancer growth
Source: Theranostics. 2020 Jan 12;10(5):2008–28. doi: 10.7150/thno.40971 (PMC7019173; doi:10.7150/thno.40971)
Supplement: Supplementary file 1 — Supplementary figures and tables. [file thnov10p2008s1.pdf]

SUPPLEMENTARY INFORMATION

Figure S1

A

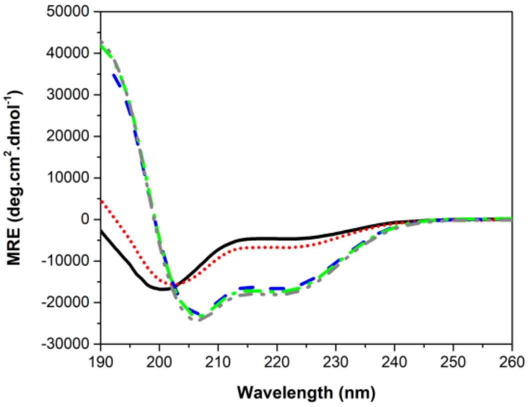

% TFE	% Helix
0	14
10	20
25	48
50	50
75	52

B **DrugScore PPI Analysis of CDK4**

Residue	ΔΔ*G* (Kcal/mol)	Residue	ΔΔ*G* (Kcal/mol)	Residue	ΔΔ*G* (Kcal/mol)
G_{48}	≤ 0	R_{55}	0.59	R_{62}	0.34
L_{49}	1.42	E_{56}	≤ 0	L_{63}	≤ 0
P_{50}	≤ 0	V_{57}	0.65	E_{64}	0.22
I_{51}	1.07	A_{58}	≤ 0	A_{65}	≤ 0
S_{52}	≤ 0	L_{59}	0.21	F_{66}	0.23
T_{53}	0.11	L_{60}	≤ 0	E_{67}	0.24
V_{54}	1.04	R_{61}	1.04		

Computational alanine scanning (Drugscore PPI) of the C-helix of CDK4 ($G_{48}L_{49}P_{50}I_{51}S_{52}T_{53}V_{54}R_{55}E_{56}V_{57}A_{58}L_{59}L_{60}R_{61}R_{62}L_{63}E_{64}A_{65}F_{66}E_{67}$) in interaction with cyclin D1 (PDB: 2W9Z). Free energy binding differences ($\Delta\Delta G$) for wildtype residue-to-Ala mutations. Positive free energy binding differences indicate a potential hot spot residue. In red $\Delta\Delta G$ >0.5 Kcal/mol, in green $\Delta\Delta G \leq 0$.

FIGURE S1 –

(A) Tendency of the peptide derived from CDK4 C helix to adopt a helical conformation

100 µM CDK4-Chelix peptide in NaP 50 mM, pH 7.0, 20°C displays a random conformation, whilst TFE induces helicity, with a maximum achieved with 25% TFE, indicating its propensity to form a helix in the appropriate environment. Overlay of far-UV CD spectra of CDK4-Chelix peptide short alone (black line) and in presence of 2,2,2-Trifluoroethanol (TFE), 10 % TFE (red dots), 25 % (blue dashed line), 50 % (green dashed-dotted line) and 75 % (grey dashed-dot-dot).

(B) Computational alanine scanning (Drugscore PPI) of the C-helix of CDK4 ($G_{48}L_{49}P_{50}I_{51}S_{52}T_{53}V_{54}R_{55}E_{56}V_{57}A_{58}L_{59}L_{60}R_{61}R_{62}L_{63}E_{64}A_{65}F_{66}E_{67}$) in interaction with cyclin D1 (PDB: 2W9Z). Free energy binding differences ($\Delta\Delta G$) for wildtype residue-to-Ala mutations. Positive free energy binding differences indicate a potential hot spot residue. In red $\Delta\Delta G$ >0.5 Kcal/mol, in green $\Delta\Delta G \leq 0$.

Figure S2

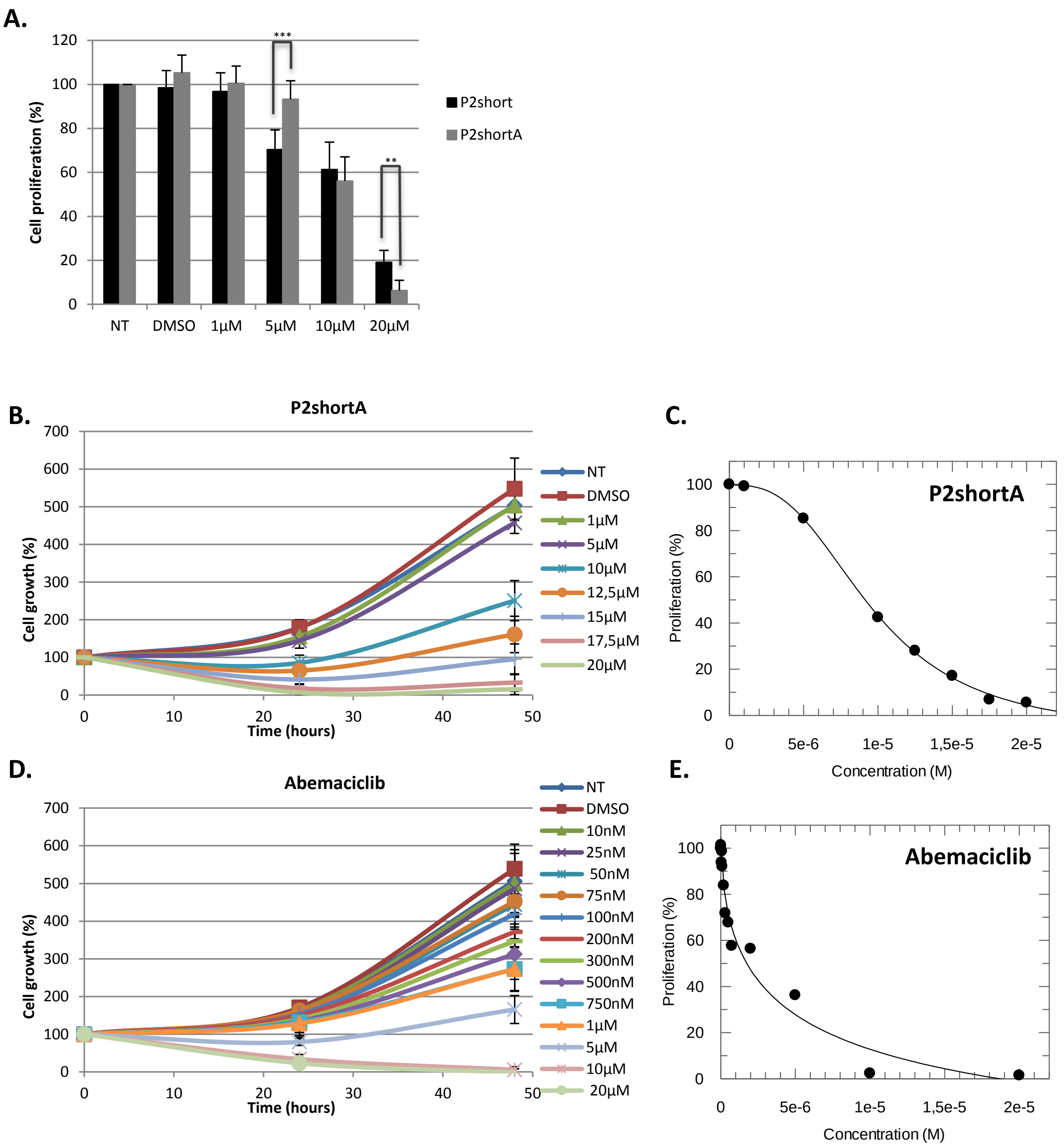

FIGURE

S2– Proliferation assays and determination of IC50s

Proliferation assays were performed by crystal violet staining every 24 hours.

(A) A549 cells were exposed to different concentrations of P2short and P2shortA peptides during 48 hours. Results are presented as average of nine independent experiments (n=4/experiment) ± SD. Means were compared with t test, * p<0,01, ** p<0,001, *** p<0,0001.

(B-G) A549 cells were treated with different concentrations of P2shortA (B,C) or Abemaciclib (D,E) for 48 hours. Curves represented means of at least two independent experiments (n=4/experiment) ± SD. IC_{50} were determined with Grafit software.

Figure S3

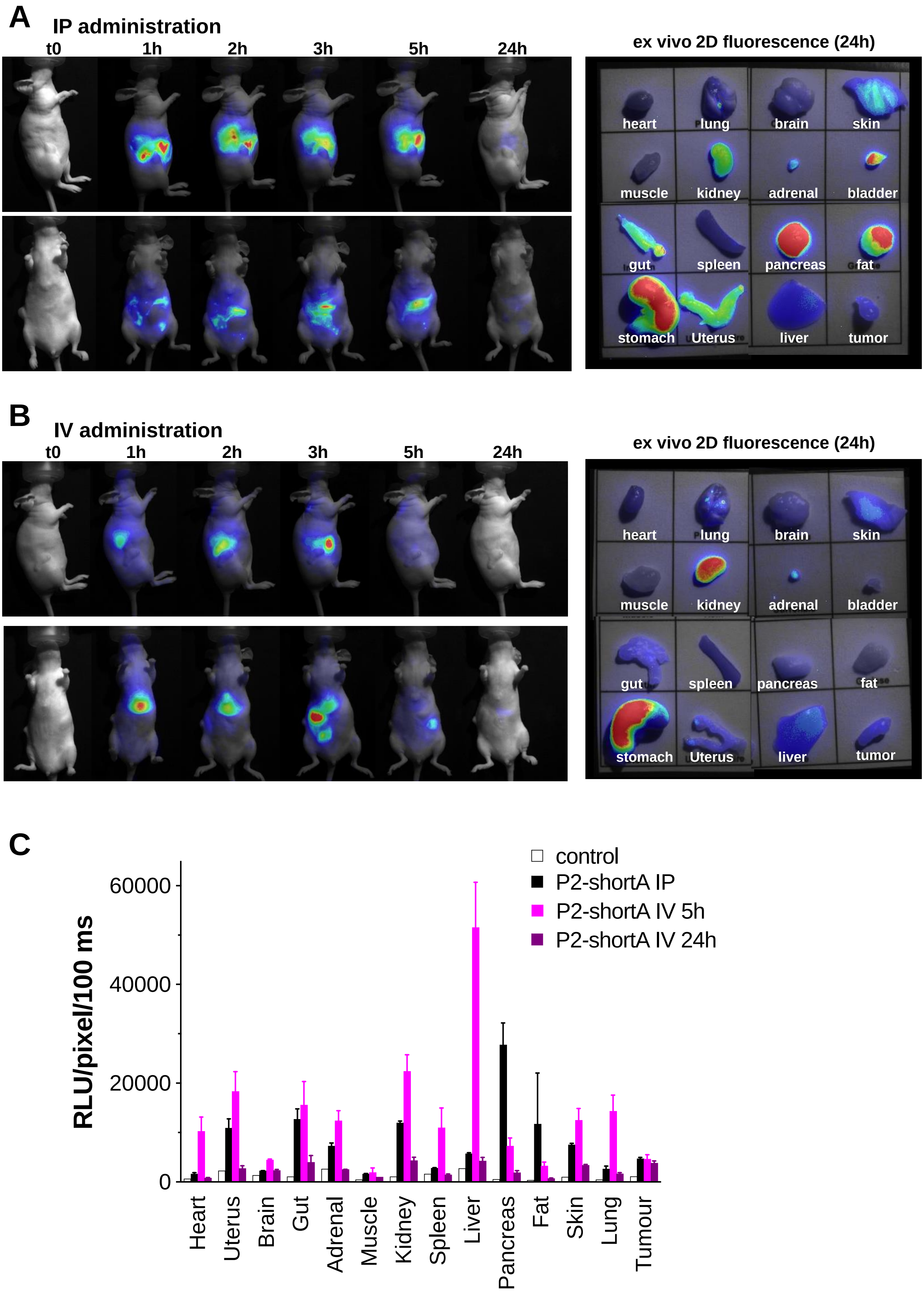
A
IP administration
t0
1h
2h
3h
5h
24h
ex vivo 2D fluorescence (24h)
heart
lung
brain
skin
muscle
kidney
adrenal
bladder
gut
spleen
pancreas
fat
stomach
Uterus
liver
tumor
B
IV administration
t0
1h
2h
3h
5h
24h
ex vivo 2D fluorescence (24h)
heart
lung
brain
skin
muscle
kidney
adrenal
bladder
gut
spleen
pancreas
fat
stomach
Uterus
liver
tumor
C
RLU/pixel/100 ms
60000
40000
20000
0
control
P2-shortA IP
P2-shortA IV 5h
P2-shortA IV 24h
Heart
Uterus
Brain
Gut
Adrenal
Muscle
Kidney
Spleen
Liver
Pancreas
Fat
Skin
Lung
Tumour

FIGURE S3 – Cy5.5-P2shortA circulates a few hours in the bloodstream after intravenous administration, but not after intraperitoneal administration, in mice with NSCLC subcutaneous tumour. 8 µmol.L^{-1} Cy5.5-P2shortA were administered intraperitoneally (IP) or intravenously (IV) in mice with subcutaneous NSCLC A549 or H358 tumour. **(A)** Fluorescence images (20 ms integration time) were recorded at the indicated time after IP injection of Cy5.5-P2-short (min-max: side 4000-23093; frontal 4000-60775). Representative images performed on isolated organs 24h after IP are shown. **(B)** Fluorescence images (20 ms integration time) were recorded at the indicated time after IV injection of Cy5.5-P2-short (min-max: side 2121-40518; frontal 2121-37520). Representative images performed on isolated organs 24h after IV injection are shown. **(C)** Region of interest (ROI) are defined on extracted organs to semi-quantify the amount of photons detected per pixel after a 100 ms exposure. The results in each organ are expressed as the mean ± SD in tumour-bearing mice (IP, n≥2; IV, n=3), or non-injected healthy mouse (control). IP injection showed biodistribution of Cy5.5-P2shortA mainly in the abdomen, whereas IV injection showed biodistribution of Cy5.5-P2shortA in several organs after 5 h and mainly in the kidneys after 24h.

Figure S4

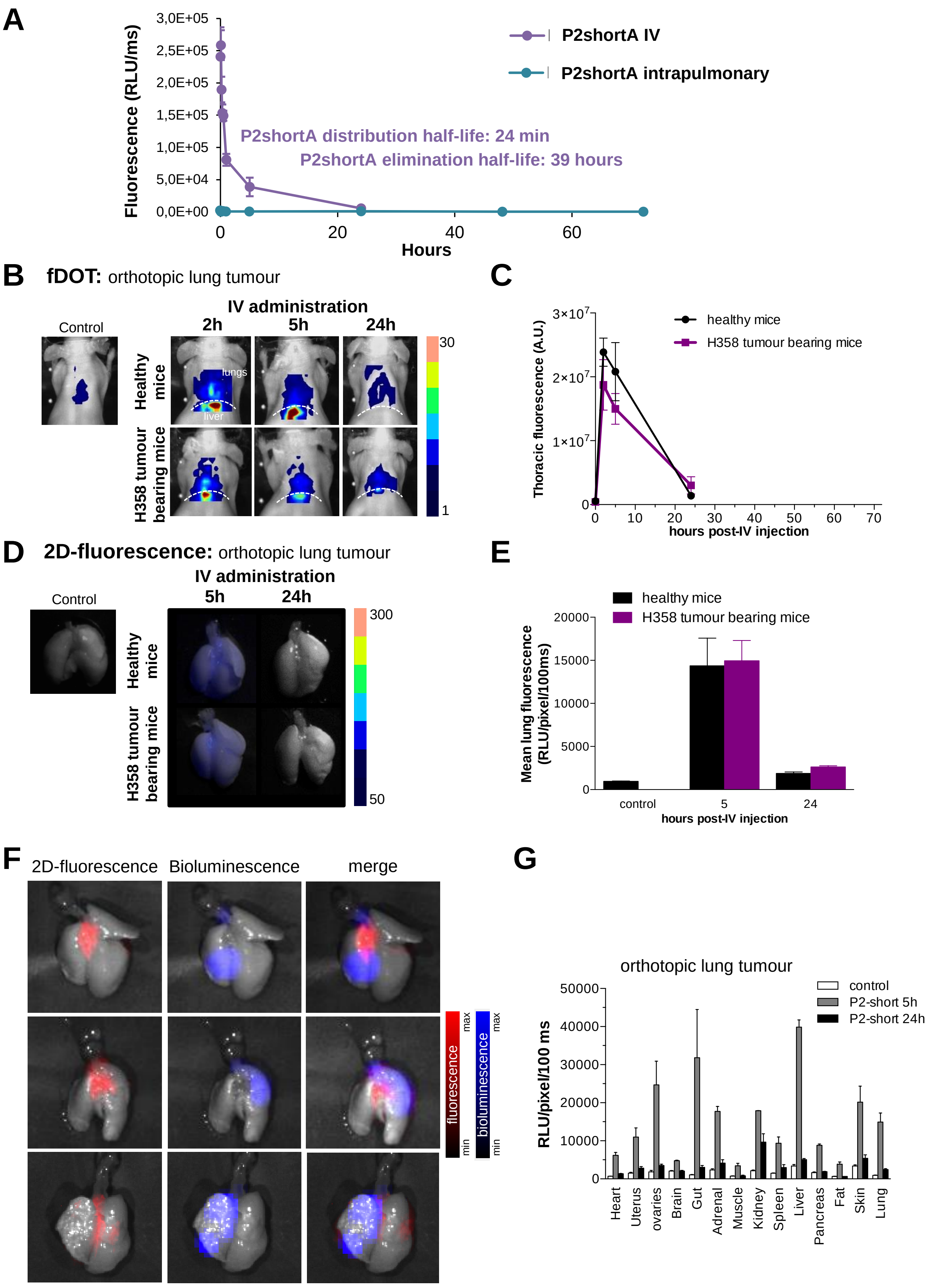
A
Fluorescence (RLU/ms)
3,0E+05
2,5E+05
2,0E+05
1,5E+05
1,0E+05
5,0E+04
0,0E+00
0
20
40
60
Hours
P2shortA IV
P2shortA intrapulmonary
P2shortA distribution half-life: 24 min
P2shortA elimination half-life: 39 hours
B
fDOT: orthotopic lung tumour
Control
IV administration
2h
5h
24h
Healthy mice
H358 tumour bearing mice
lungs
liver
30
1
C
Thoracic fluorescence (A.U.)
healthy mice
H358 tumour bearing mice
hours post-IV injection
D
2D-fluorescence: orthotopic lung tumour
Control
IV administration
5h
24h
Healthy mice
H358 tumour bearing mice
300
50
E
Mean lung fluorescence (RLU/pixel/100ms)
healthy mice
H358 tumour bearing mice
20000
15000
10000
5000
0
control
5
24
hours post-IV injection
F
2D-fluorescence
Bioluminescence
merge
fluorescence
bioluminescence
max
min
G
orthotopic lung tumour
RLU/pixel/100 ms
50000
40000
30000
20000
10000
0
control
P2-short 5h
P2-short 24h
Heart
Uterus
ovaries
Brain
Gut
Adrenal
Muscle
Kidney
Spleen
Liver
Pancreas
Fat
Skin
Lung

FIGURE S4 – Cy5.5-P2shortA circulates in the bloodstream after intravenous injection.

(A) Healthy mice were injected intravenously (200µL) or intrapulmonary (50µL) with 10 µmol.L^{-1} Cy5.5-P2shortA. The fluorescence intensity measurements of blood samples were performed over time. The results are expressed as the mean ± SD (n=6). Intravenous injection showed Cy5.5-P2shortA blood distribution half-life of 24 min and Cy5.5-P2shortA blood elimination half-life of 39h, whereas intrapulmonary administration showed Cy5.5-P2shortA below sensitivity threshold. **(B-G)** 10 µmol.L^{-1} Cy5.5-P2shortA were administered intravenously (200 µL) in orthotopic H358 tumour-bearing mice. **(B)** 3D-fluorescence tomography imaging (fDOT) was performed at the indicated time after administration of Cy5.5-P2shortA. Dotted line: position of diaphragm. **(C)** Volumes of interest are defined on the thoracic region to semi-quantify the total amount of photons. The results are expressed as the mean ± SEM in healthy (5h, n≥5; 24h, n≥3) or H358 tumour-bearing mice (5h, n≥6; 24h, n≥2). **(D)** Fluorescent images were performed on isolated lungs at the indicated time after Cy5.5-P2shortA injection. **(E)** Regions of interest (ROIs) are defined on the extracted lungs to semi-quantify the amount of photons detected per pixel after a 100 ms exposure. The results are expressed as the mean ± SEM in healthy (n=4), or H358 tumour-bearing lungs (n≥3), or non-injected healthy mouse (control). **(F)** Bioluminescence, and 2D-fluorescence imaging were performed on isolated lungs 5h after intravenous injection of Cy5.5-P2shortA. Bioluminescent signal showed H358-Luc tumour cells in lungs (in blue); 2D-fluorescent signal showed Cy5.5-P2shortA location in the lungs (in red); fluorescent and bioluminescent signals were merge. No colocalization between Cy5.5-P2shortA signal and H358 tumours was observed in lungs after intravenous injection of P2-short. **(G)** ROIs are defined on the organs to semi-quantify the amount of photons detected per pixel after a 100 ms exposure. The results are expressed as the mean ± SEM in healthy (n=4), or H358 tumour-bearing lungs (n≥3), or non-injected healthy mouse (control).

Figure S5

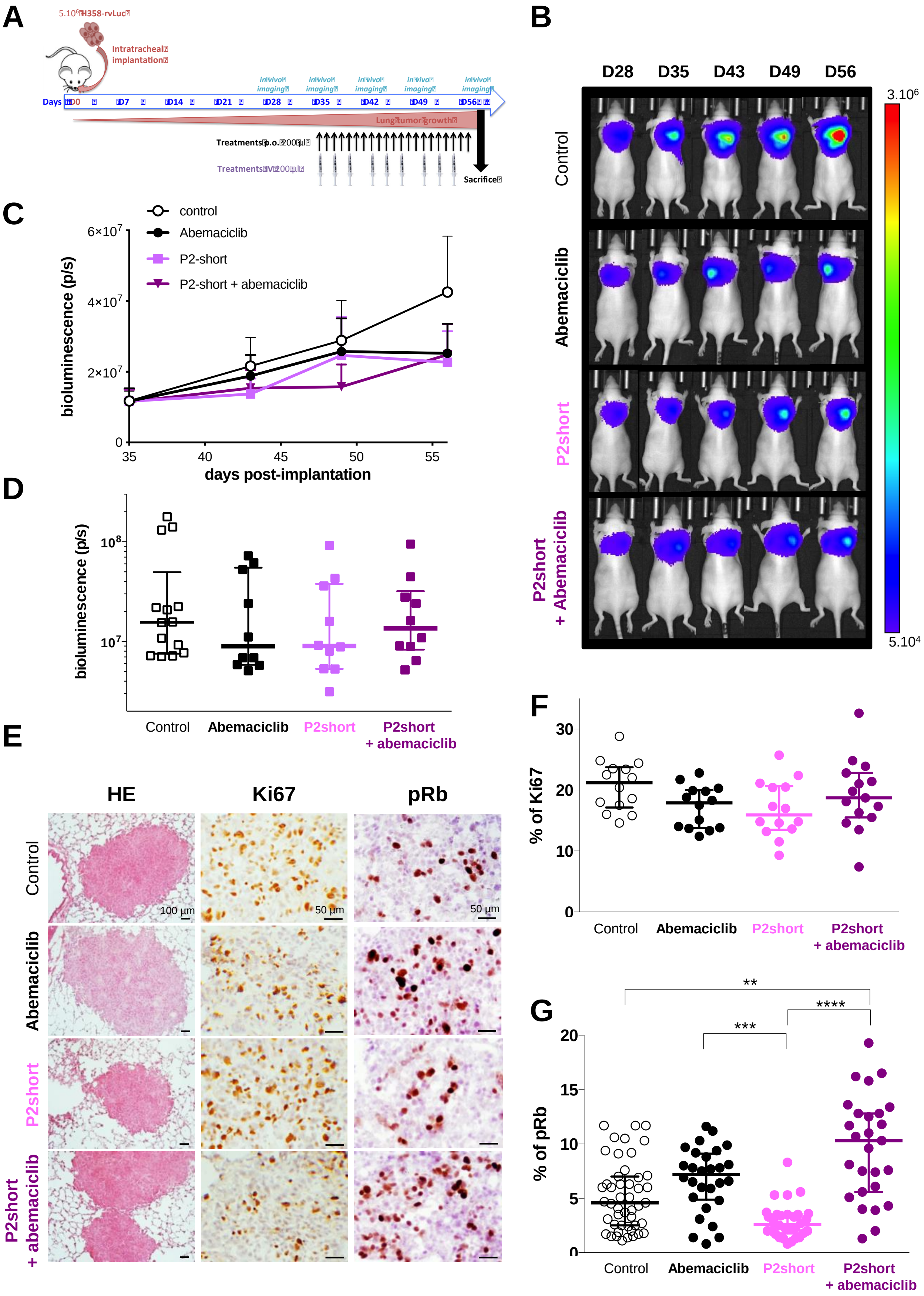
A
5.10⁶ H358-rvLuc
Intratracheal implantation
Days D0 D7 D14 D21 D28 D35 D42 D49 D56
in vivo imaging
Lung tumor growth
Treatments p.o. 200 µl
Treatments IV 200 µl
Sacrifice
B
D28 D35 D43 D49 D56
Control
Abemaciclib
P2short
P2short + Abemaciclib
3.10⁶
5.10⁴
C
control
Abemaciclib
P2-short
P2-short + abemaciclib
bioluminescence (p/s)
days post-implantation
D
bioluminescence (p/s)
Control
Abemaciclib
P2short
P2short + abemaciclib
E
HE
Ki67
pRb
Control
Abemaciclib
P2short
P2short + abemaciclib
100 µm
50 µm
F
% of Ki67
Control
Abemaciclib
P2short
P2short + abemaciclib
G
% of pRb
**
***
****
Control
Abemaciclib
P2short
P2short + abemaciclib

FIGURE S5 – Effect of P2shortA and Abemaciclib in orthotopic H358 tumour-bearing mice following intravenous injection

(A) Schematic representation of H358 orthotopic tumours growth and of the treatments plan. The mice were inoculated with H358-rvLuc cells and randomized after 5 weeks into 3 groups of 10 mice and one control group of 14 mice. Vehicle or Abemaciclib 10 $mg.kg^{-1}$ were administered *per os* every days for 3 weeks. 0.9 $mg.kg^{-1}$ P2shortA was injected intravenously 3 times a week for 3 weeks. **(B)** Overtime thoracic bioluminescence images of H358-Luc tumours. One representative mouse (dorsal view) per group is shown. **(C)** Treatments with abemaciclib and/or P2shortA trend to inhibit the growth of orthotopic H358 tumours. The results are expressed as the mean ± SEM (control group, n=14; treated groups, n=10). **(D)** Thoracic bioluminescence level in each mouse at day 56. Bars, medians with interquartiles. **(E)** Histological (HE) and immunohistochemistry for Ki67 and phosphorylated-Rb (pRb) on lung tumour sections. Scale bars, 100 µm (HE) and 50 µm (Ki67 and pRb). **(F)** Ki67-positive cells were quantified on different fields (2-8) in three or four mice per group, and reported as % Ki67 positive cells. Bars, medians with interquartiles. **(G)** pRb-positive cells were quantified on different fields (2-29) in three or four mice per group, and reported as % pRb positive cells. Bars, medians with interquartiles. Kruskall-Wallis test with Dunn's multiple comparisons *posthoc* tests ($p < 0.0001$); **, treatment combination group compared to control group; ***, abemaciclib group compared to P2shortA group; ****, treatment combination group compared to P2shortA group.

Figure S6

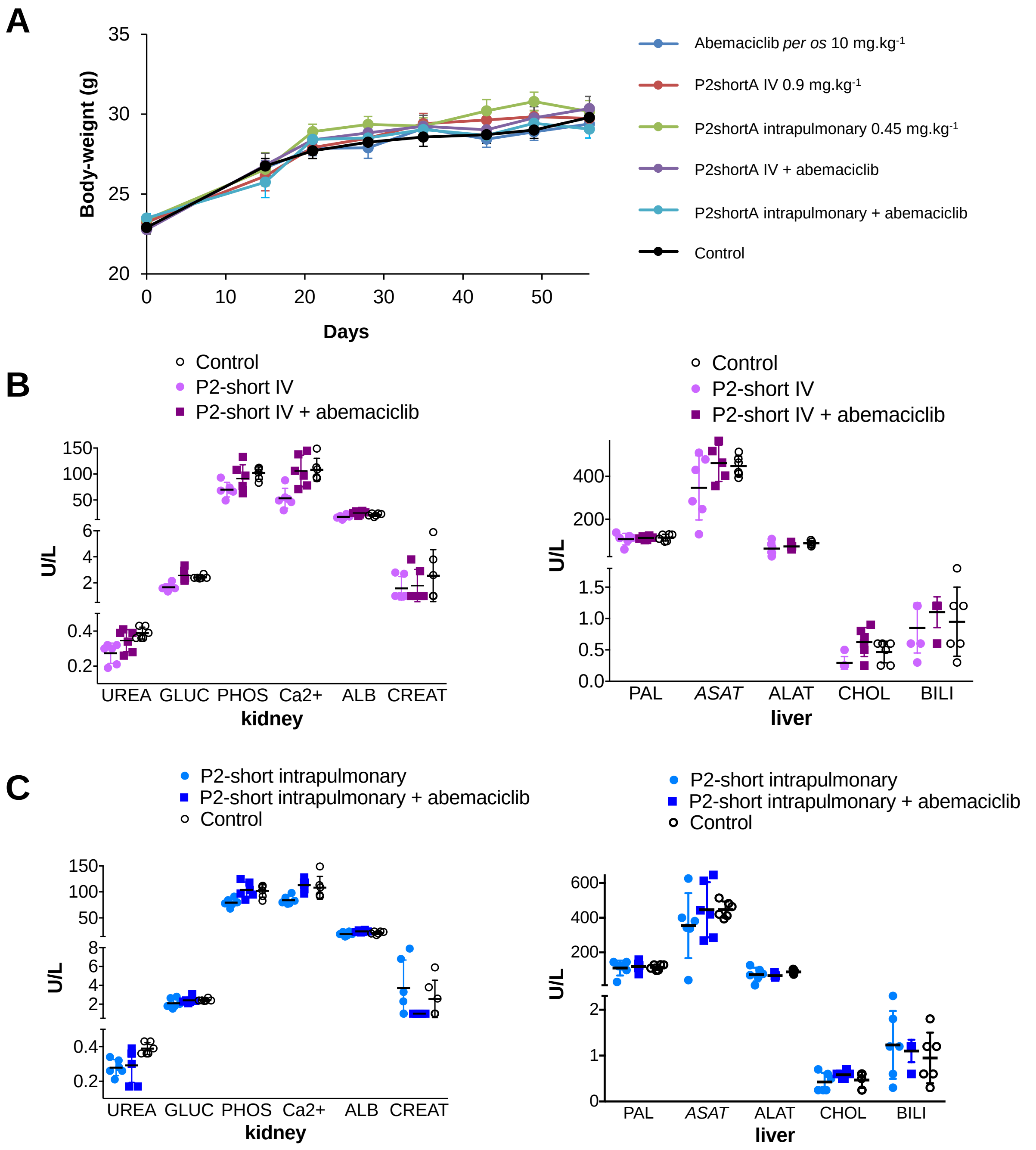

FIGURE S6 – P2shortA and Abemaciclib treatments were well-tolerated in mice with orthotopic H358 lung tumour.

(A) Mice body-weight in each group from the tumour implantation to sacrifice at day 56. Treatments began at day 35 (means ± SD; control, n=14; treated groups, n=10). **(B-C)** Plasma biochemical values of mice after intravenous (**B**) or intrapulmonary (**C**) administration of P2shortA were assessed. Data represent the means ± SD of 6 mice per group. UREA: blood urea nitrogen; GLUC, glucose; PHOS, phosphorus; Ca2+, calcium; ALB, albumin; CREAT, creatinine; PAL, alkaline phosphatase; ASAT, aspartate amino-transferase; ALAT, alanine amino-transferase; CHOL, cholesterol, BILI, bilirubin.

Table S1. Clinical and biological characteristics of patients according to the histological subtype.

	**All patients** n=215 n (%)	**ADC** n=111 n (%)	**SCC** n=104 n (%)	*p* value
**Gender**				
Male	161 (75%)	71 (64%)	90 (88%)	0.0001
Female	54 (25%)	40 (36%)	14 (12%)	
**Age**, Median (range)	61.2 (47-70)	64 (40-82)	67.5 (41-85)	
**Smoking status**				
Current smokers	102 (48%)	56 (50%)	46 (44%)	0.0069
Former smokers	95 (44%)	40 (36%)	55 (53%)	
Passive smokers	2 (1%)	1 (1%)	1 (1%)	
Non -smokers	16 (7%)	14 (13%)	2 (2%)	
Professional exposures	50 (23%)	25 (23%)	25 (24%)	
**Stage**	n=211	n=108	n=103	0.02
I	79 (38%)	42 (39%)	37 (36%)	
II	57 (27%)	24 (22%)	33 (32%)	
III	60 (28%)	29 (27%)	31 (30%)	
IV	15 (7%)	13 (12%)	2 (2%)	
EGFR* mutation	n=112	n=109	n=3	1.00
No mutation	100 (89%)	97 (89%)	3 (100%)	
Mutation (exons 18-21)	12 (11%)[#]	12 (11%)	0	
KRAS* mutation	n=112	n=108	n=4	0.30
No mutation	73 (65%)	69 (64%)	4 (100%)	
Mutation (exon 2)	39 (35%)	39 (36%)	0	
HER2* mutation	n=86	n=85	n=1	1.00
No mutation	83 (97%)	82 (96%)	1 (100%)	
Mutation	3 (3%)	3 (4%)	0	
PI3KCA* mutation	n=30	n=30	n=0	
No mutation	30 (100%)	30 (100%)		
Mutation	0	0		
**BRAF mutation***	n=89	n=86	n=3	0.13
No mutation	85 (96%)	83 (97%)	2 (67%)	
Mutation	4 (4%)	3 (3%)	1 (33%)	
FGFR1* amplification*	n=5	n=0	n=5	
No amplification	5 (100%)	0	5 (100%)	
Amplification	0	0	0	
ALK* rearrangement*	n=66	n=64	n=2	1.00
Negative	66 (100%)	64 (100%)	2 (100%)	
Positive	0	0	0	
ROS1* rearrangement*	n=30	n=27	n=3	1.00
Negative	29 (97%)	26 (96%)	3 (100%)	
Positive	1 (3%)	1 (4%)	0	
CMET* amplification*	n=11	n=8	n=3	1.00
Negative	10 (91%)	7 (88%)	3 (100%)	
Positive	1 (9%)	1 (12%)	0	
**Cyclin D1 expression**	n=206	n=107	n=99	<0.0001
0-10%	87 (42%)	24 (22%)	63 (64%)	
>10%	119 (58%)	83 (78%)	36 (36%)	
**CDK4 expression**	n=215	n=111	n=104	0.67
0-10%	81 (38%)	40 (36%)	41 (39%)	
>10%	134 (62%)	71 (64%)	63 (61%)	
**pRb expression**	n=215	n=111	n=104	0.0003
0-10%	166 (77%)	97 (87%)	69 (66%)	
>10%	49 (23%)	14 (13%)	35 (34%)	

*Mutations were detected by next- generation sequencing (NGS). **Rearrangements and amplifications were detected by immunohistochemistry and/or fluorescent in situ hybridization (FISH). Statistical analysis was

performed using Fisher's exact test. Missing data were excluded from the analysis. #One mutation of exon 18, five deletions of exon 19, one mutation of exon 20, and five substitution of exon 21 L858R.

Table S2: immunohistochemical and molecular characterization of NSCLC tumours

	Cyclin D1 expression				CDK4 expression				pRb expression		
	All patients	0-10%	>10%		All patients	0-10%	>10%		0-10%	>10%	
		n=87	n=119			n=81	n=134		n=166	n=49	
	n (%)	n (%)	n (%)	*p* value	n (%)	n (%)	n (%)	*p* value	n (%)	n (%)	*p* value
**Cyclin D1 expression**	n=206				n=206			0.0001			0.9
0-10%	87 (42%)				87 (42%)	47 (54%)	40 (46%)		66 (76%)	21 (34%)	
>10%	119 (58%)				119 (58%)	29 (24%)	90 (76%)		91 (76%)	28 (24%)	
**CDK4 expression**	n=206			0.0001	n=215						
0-10%	76 (37%)	47 (54%)	29 (24%)		81 (38%)				72 (89%)	9 (11%)	0.0014
>10%	130 (63%)	40 (46%)	90 (76%)		134 (62%)				94 (70%)	40 (30%)	
**pRb expression**	n=206			0.9	n=215			0.0014			
0-10%	157 (76%)	66 (76%)	91 (76%)		166 (77%)	72 (89%)	94 (70%)				
>10%	49 (24%)	21 (34%)	28 (24%)		49 (23%)	9 (11%)	40 (30%)				
EGFR	n=108	n=26	n=82	1.00	n=112	n=41	n=71	0.53	n=96	n=16	0.20
No mutation	97 (90%)	24 (72%)	73 (89%)		100 (89%)	38 (93%)	62 (87%)		84 (88%)	16 (100%)	
Mutation [a]	11 (10%)	2 (8%)	9 (11%)		12 (11%)	3 (7%)	9 (13%)		12 (12%)	0	
KRAS	n=107	n=26	n=81		n=112	n=41	n=71		n=97	n=15	
No mutation	69 (64%)	22 (85%)	47 (58%)	0.0178	73 (65%)	25 (61%)	48 (68%)	0.53	62 (64%)	11 (73%)	0.57
Mutation [b]	38 (36%)	4 (15%)	34 (42%)		39 (35%)	16 (39%)	23 (32%)		35 (36%)	4 (27%)	
HER2	n=81	n=19	n=62	1.00	n=83	n=30	n=53	1.00	n=73	n=10	0.32
No mutation	78 (96%)	19 (100%)	59 (95%)		80 (96%)	29 (97%)	51 (96%)		71 (97%)	9 (90%)	
Mutation	3 (4%)	0	3 (5%)		3 (4%)	1 (3%)	2 (4%)		2 (3%)	1 (10%)	
PI3KCA	n=28	n=7	n=21		n=31	n=9	n=22		n=28	n=3	
No mutation	28 (100%)	7 (100%)	21 (100%)		31 (100%)	9 (100%)	22 (100%)		28 (100%)	3 (100%)	
Mutation	0	0	0		0	0	0		0	0	
BRAF	n=85	n=20	n=65	1.00	n=88	n=32	n=56	0.0154	n=77	n=11	1.00
No mutation	83 (98%)	20 (100%)	63 (97%)		84 (95%)	28 (88%)	56 (100%)		73 (95%)	11 (100%)	
Mutation	2 (2%)	0	2 (3%)		4 (5%)	4 (12%)	0		4 (5%)	0	
FGFR1	n=5	n=2	n=3		n=5	n=1	n=4		n=4	n=1	
No amplification	5 (100%)	2 (100%)	3 (100%)		5 (100%)	1 (100%)	4 (100%)		4 (100%)	1 (100%)	
Amplification	0	0	0		0	0	0		0	0	
ALK [c]	n=62	n=10	n=52		n=66	n=18	n=48		n=61	n=5	
Negative	62 (100%)	10 (100%)	52 (100%)		66 (100%)	18 (100%)	48 (100%)		61 (100%)	5 (100%)	
Positive	0	0	0		0	0	0		0	0	
ROS1 [c]	n=29	n=2	n=27	1.00	n=30	n=7	n=23	1.00	n=29	n=1	1.00
Negative	28 (97%)	2 (100%)	26 (96%)		29 (97%)	7 (100%)	22 (96%)		28 (97%)	1 (100%)	
Positive	1 (3%)	0	1 (4%)		1 (3%)	0	1 (4%)		1 (3%)	0	
CMET	n=10	n=1	n=9	1.00	n=11	n=3	n=8	1.00	n=11	n=0	1.00
No amplification	9 (90%)	1 (100%)	8 (89%)		10 (91%)	3 (100%)	7 (88%)		10 (91%)	0	
Amplification	1 (10%)	0	1 (11%)		1 (9%)	0	1 (12%)		1 (9%)	0	

Missing data were excluded from the analysis. [a] exons 18-21; [b] exon 2; [c] rearrangement. Statistical analysis was performed using Fisher's exact test
